# IP6‐stabilised HIV capsids evade cGAS/STING‐mediated host immune sensing

**DOI:** 10.15252/embr.202256275

**Published:** 2023-03-27

**Authors:** Guido Papa, Anna Albecka, Donna Mallery, Marina Vaysburd, Nadine Renner, Leo C James

**Affiliations:** ^1^ MRC Laboratory of Molecular Biology, Protein & Nucleic Acid Division Cambridge UK

**Keywords:** capsid stability, DNA sensing, HIV, innate immunity, IP6, Immunology, Microbiology, Virology & Host Pathogen Interaction, Signal Transduction

## Abstract

HIV‐1 uses inositol hexakisphosphate (IP6) to build a metastable capsid capable of delivering its genome into the host nucleus. Here, we show that viruses that are unable to package IP6 lack capsid protection and are detected by innate immunity, resulting in the activation of an antiviral state that inhibits infection. Disrupting IP6 enrichment results in defective capsids that trigger cytokine and chemokine responses during infection of both primary macrophages and T‐cell lines. Restoring IP6 enrichment with a single mutation rescues the ability of HIV‐1 to infect cells without being detected. Using a combination of capsid mutants and CRISPR‐derived knockout cell lines for RNA and DNA sensors, we show that immune sensing is dependent upon the cGAS–STING axis and independent of capsid detection. Sensing requires the synthesis of viral DNA and is prevented by reverse transcriptase inhibitors or reverse transcriptase active‐site mutation. These results demonstrate that IP6 is required to build capsids that can successfully transit the cell and avoid host innate immune sensing.

## Graphical abstract

HIV‐1 uses IP6 to build a capsid inside which it can synthesise DNA while avoiding detection by cGAS/STING‐mediated DNA sensing. Capsids lacking IP6 become unstable, expose viral DNA and alert host immunity.

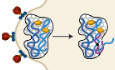

## Introduction

HIV is an enveloped retrovirus with two copies of single‐stranded positive‐sense RNA genome packaged inside a proteinaceous cone‐shaped capsid core. The HIV capsid is a metastable structure, capable of surviving prolonged exposure in the cytosol but also of efficiently disassembling, termed “uncoating,” prior to integration. The metabolite inositol hexakisphosphate (IP6) has recently been proposed as a key cofactor that drives HIV capsid formation and maintains its stability (Mallery *et al*, 2018; Dick *et al*, 2018a,b). IP6 is an abundant polyanion found in all cells and is selectively packaged and enriched into HIV virions when they bud from the cell (Mallery *et al*, 2019). In the first step of HIV virion assembly, IP6 is recruited into budding virions by the immature Gag lattice via a binding site at the centre of immature hexamers formed by two charged lysine rings—Lys158 (K158) and Lys227 (K227) (Mallery *et al*, 2019). A single IP6 molecule per immature hexamer is bound above a six‐helix bundle (6HB) that forms at the junction between the CA and spacer peptide 1 (SP1) domains of Gag. Upon budding, the 6HB is cleaved by the viral protease leading to the disassembly of the immature lattice and the release of IP6 inside the virion (Dick *et al*, 2018b). Liberated CA protein then assembles into hexamers and 12 pentamers that together form the mature conical capsid. IP6 is essential during this process of mature capsid formation (Renner *et al*, 2023). Mutation of either Gag lysine responsible for IP6 binding (K158A or K227A) results in loss of IP6‐packaging into virions, defective capsid formation and a profound loss of infectivity (Mallery *et al*, 2019). Importantly, a second‐site mutation in the SP1 domain, T8I, rescues K158A or K227A by stabilising the 6HB and enabling the single remaining lysine ring to package sufficient IP6 for virus release and mature capsid formation (Mallery *et al*, 2021).

Forming a proper capsid and maintaining it after cell entry is essential for HIV infection. This is because HIV uses the capsid as an interactive platform to recruit host cofactors for cellular transport and nuclear entry (Wilen *et al*, 2012; Dick *et al*, 2018a; James, 2019). The capsid also protects the viral genome from host innate immune sensors during the early stage of infection (James, 2019; Sumner *et al*, 2020; Yin *et al*, 2020). Sensors such as cGAS can detect exposed DNA and drive inflammatory signalling (Mankan *et al*, 2014). By carrying out DNA synthesis inside the capsid, HIV‐1 can prevent cGAS detection. However, this requires the capsid to be permeable to nucleotides to fuel synthesis and to remain intact during the process of reverse transcription. We have previously proposed that both requirements are fulfilled by the hundreds of charged pores that form at the centre of the hexamers and pentamers that make up the capsid lattice. These charged pores bind nucleotides, increasing the local concentration and promoting encapsidated DNA synthesis (Jacques *et al*, 2016). They also bind IP6, which greatly increases hexamer and capsid stability (Mallery *et al*, 2018, 2019; Dick *et al*, 2018a; Renner *et al*, 2021). In the absence of IP6, capsids collapse within minutes, whereas in the presence of IP6, they are stable for many hours. Moreover, IP6 stabilises the capsid during the process of reverse transcription: During encapsidated DNA synthesis, capsids lacking IP6 collapse before reverse transcription is completed (Mallery *et al*, 2018; Christensen *et al*, 2020; Jennings *et al*, 2020).

We hypothesised that if IP6 is necessary for capsids to form and to remain intact during reverse transcription then HIV‐1 may be dependent upon the metabolite to avoid innate immune signalling. Here we show that HIV‐1 viruses that are unable to enrich IP6 into virions are sensed by cGAS during infection of macrophages and T‐cell lines. Conversely, restoring IP6 enrichment rescues the ability of HIV‐1 to evade immune detection. Our results demonstrate that IP6 is not only important to promote HIV‐1 infection but also to avoid host responses.

## Results and Discussion

### IP6‐deficient HIV‐1 capsid triggers an ISG response in macrophages cell line

**Figure 1:**
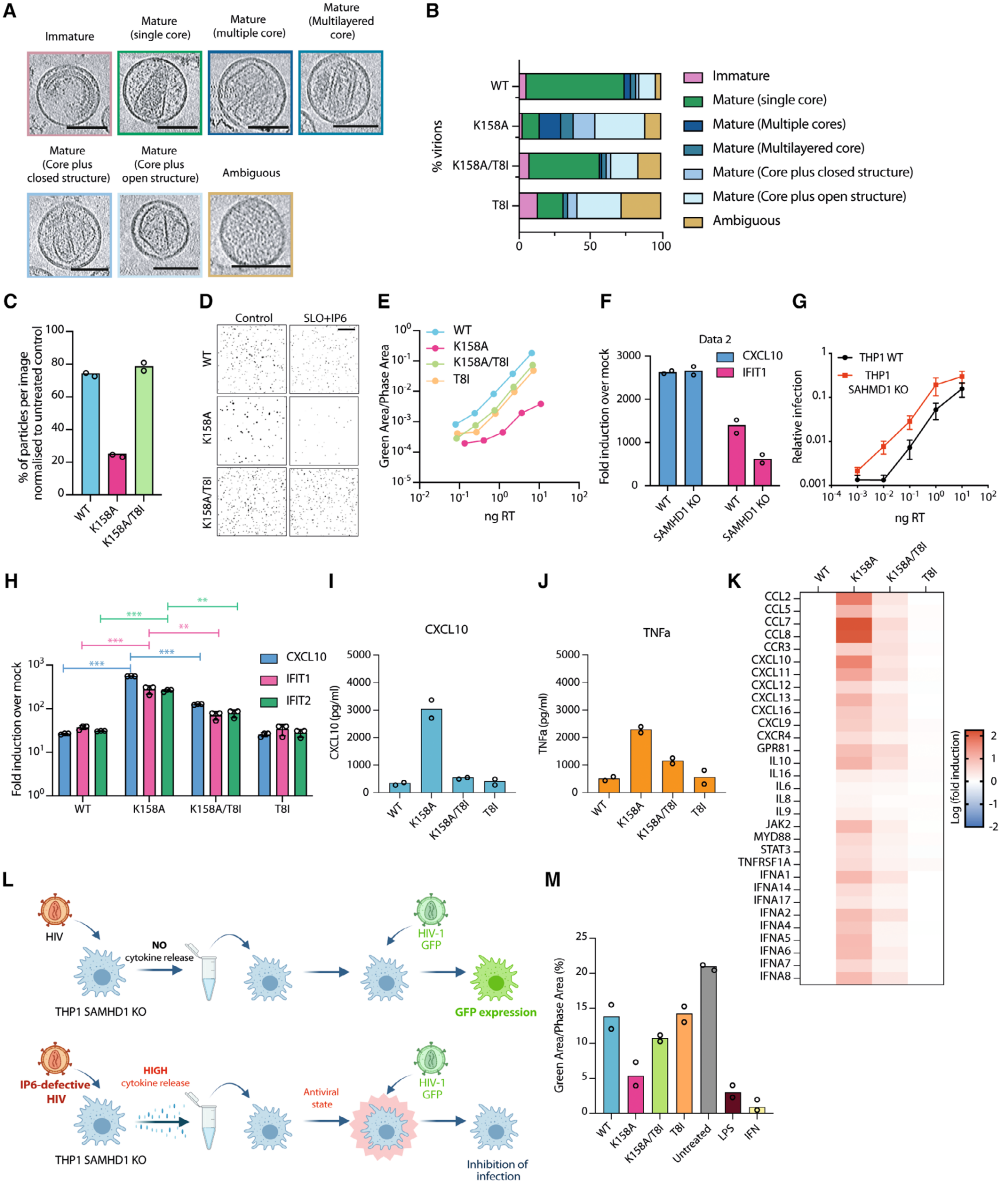
IP6‐deficient HIV‐1 capsid triggers an ISG response in macrophage cell line A, B
Capsids for WT, K158A, K158A/T8I and T8I were classified into the indicated categories. Representative tomograms of K158A mutant (A) and the percentage (B) for each category are shown. The margin of error was calculated as previously described (Mallery *et al*, 2021) and is shown in Fig EV1A. Images and quantification are from the same samples. Scale bars: 100 nm.C
Virion stability assay using TIRF microscopy to quantify the number of viral particles in presence of SLO and IP6 for the indicated mutant after 1 h. Mean data represent *n* = 2 technical replicates. Scale bar 10 μm.D
Representative images of the quantification in (C). Scale bar 10 μm.E
Titration of virus onto HEK293T cells with the indicated ng/RT of the different HIV‐1 capsid mutant expressing GFP. Infection is quantified as GFP Area/Phase Area normalised to Mock. Mean data represent *n* = 2 biological replicates.F
Quantification of CXCL10 and IFIT1 transcripts by qPCR of the indicated THP‐1 cell lines stimulated for 24 h with LPS (1 ng/ml) normalised to mock. Mean data *n* = 2 biological replicates.G
Titration of virus onto THP‐1 WT and THP‐1‐SAMHD1 KO cells with the indicated ng/RT of the different HIV‐1 capsid mutant expressing GFP. Infection is quantified as GFP Area/Phase Area normalised to Mock. Mean data represent *n* = 2 biological replicates.H
Quantification of the indicated chemokine transcripts by qPCR from PMA‐treated THP‐1/SAMHD1 KO cells transduced for 24 h with 50 ng/RT of the indicated HIV‐1 GFP capsid mutants normalised to mock‐infected. Mean data represent *n* = 3 biological replicates.I, J
Protein quantification of CXCL10 (I) and TNFa (J) by Luminex™ from the supernatant of PMA‐treated THP‐1/SAMHD1 KO cells infected with the indicated HIV‐1 mutants. Data were normalised to mock. Mean data represent *n* = 2 biological replicates.K
Quantification of the indicated immune transcripts performed by qPCR on PMA‐treated THP‐1/SAMHD1 KO cells infected with the indicated HIV‐1 mutants. Data are shown as Log fold induction over WT HIV‐1. Mean data represent *n* = 3 biological replicates.L
Schematic representation of media conditioning experiment. The media from cells infected with WT or IP6‐deficient HIV‐1 was transferred onto fresh THP‐1‐SAMHD1 KO cells. WT HIV‐1 expressing GFP was then added to the fresh cells 18 h post media transfer and infection was monitored by GFP expression. In the case of a strong immune response, cytokines present in the media would inhibit infection of the WT HIV‐1 GFP virus. Created with BioRender.com.M
Relative infection shown as Green Area/Phase Area of PMA‐treated THP‐1/SAMHD1 KO cells prestimulated with media from cells infected with the indicated HIV‐1 mutants or the indicated conditions. LPS (1 ng/ml) and IFN (1 ng/ml) were shown as a positive control. Mean data represent *n* = 2 biological replicates. Data information: Unless otherwise indicated, mean data represent *n* = 2 biological replicates. Error bars depict the mean ± SEM. Statistical analyses were performed with the Student's *t* test *P < 0.05, **P < 0.01, ***P < 0.001, *n.s*. = not significant.

**Figure EV1:**
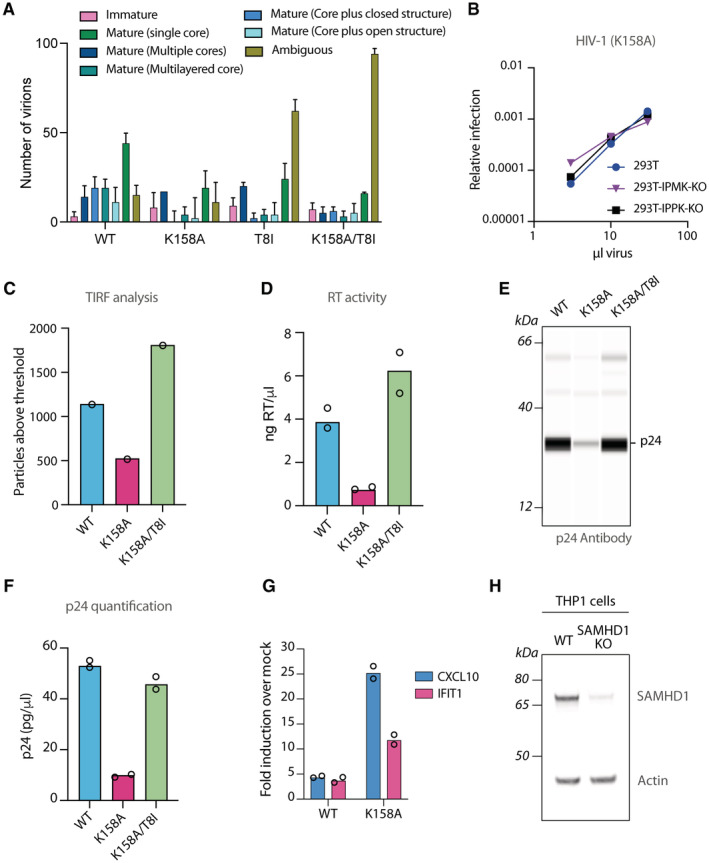
Classification of HIV‐1 tomograms and quantification and stability of HIV mutants A
Number of virions per category for each mutant, with error bars showing the margin of error (MOE; see Materials and Methods).B
Titration of virus onto 293T cells with the indicated ng/RT of the HIV‐1 capsid mutant K158A expressing GFP. Infection is quantified as GFP Area/Phase Area normalised to Mock.C
Quantification of the indicated HIV mutant virions using TIRF microscopy.D
RT activity quantification of the indicated HIV mutants.E, F
Capillary western blot for p24 of HIV produced with the indicated capsid mutants (E) and p24 quantification (F).G
Quantification of the indicated chemokine transcripts by qPCR from PMA‐treated THP‐1 WT cells transduced for 24 h with 50 ng/RT of the indicated HIV‐1 GFP capsid mutants.H
SAMHD1 western blot of THP‐1 WT and SAMHD1 KO cells. Actin was used as a loading control. Data information: All mean data represent *n* = 2 biological replicates. Source data are available online for this figure.

To investigate whether capsid formation and stabilisation by IP6 is important for HIV‐1 to escape immune sensing, we decided to compare the immature capsid mutant K158A, which is unable to enrich IP6 into assembling virions (Mallery *et al*, 2019), with the double mutant K158A/T8I where IP6‐packaging is restored (Mallery *et al*, 2021). We therefore produced a matched panel of viruses including WT, K158A, K158A/T8I and T8I and characterised them in parallel cryoET, TIRF microscopy and infection assays. Three‐dimensional (3D) tomographic reconstructions confirmed that the previously described IP6‐deficient mutant K158A undergoes maturation inefficiently and forms capsids with additional open structures (Mallery *et al*, 2021; Fig 1A). K158A virions with aberrant structures form a majority (> 70%) of the population, unlike WT virions, which mostly contain a single closed core (Fig 1B). The frequency of aberrant capsids was substantially reduced by the addition of the T8I mutation in the SP1 portion of capsid protein (K158A/T8I; Figs 1B and EV1A), consistent with previous reports showing that this mutation rescues capsid formation by restoring the ability of the immature lattice to recruit IP6 into virions (Mallery *et al*, 2021).

Previously published data suggest that K158A capsids are less stable than WT (Mallery *et al*, 2021). We therefore tested our panel of WT, K158A and K158A/T8I virions for capsid stability using a modified Total Internal Reflection Fluorescence (TIRF) microscopy assay to measure capsid persistence in presence of IP6. Virions were treated with Streptolysin‐O (SLO), which makes pores in the lipid envelope to simulate capsid exposure, and IP6 was added. The number of capsid particles remaining after 1 h was quantified using anti‐p24 antibodies. Most (~ 80%) K158A capsids collapsed during this time (Fig 1C and D). Of note, when the T8I stabilising mutation was added to the K158A mutant, the number of capsid particles recovered to WT levels, suggesting that T8I can restore capsid stability. Together with the cryoET data, these data suggest that K158A produces defective capsids that are intrinsically less stable than WT.

Parallel infection experiments performed with the same virions showed that capsid defects introduced by K158A are associated with a profound loss of infection (Fig 1E). Additionally, the low infectivity of the K158 mutant was not affected by the removal of IP6 from target cells lacking IPPK or IPMK enzymes (Fig EV1B; Mallery *et al*, 2019), suggesting that the concentration of IP6 is still well above that of the capsid K_D_. To assess the relative contribution of changes in viral production and viral infectivity to the decreased level of infection, we assessed production using three independent assays: (i) number of particles using TIRF microscopy (Fig EV1C); (ii) reverse transcriptase (RT) activity (Fig EV1D); (iii) amount of p24 capsid protein (Fig EV1E and F). All three measurements showed a similar difference (~ 3–5 fold) in viral production between WT, K158A and K158A/T8I, with a particularly close correspondence between p24 and RT measurements, both of which are routinely used as methods for normalising infection. Given that production is only modestly affected by K158A, this suggests that the most substantial deleterious impact of this mutation is on infectivity and that this is the principal cause of the poor levels of K158A infection.

Next, we took this panel of characterised viruses and used them to infect monocytic THP‐1 cells, which can be differentiated by treatment with phorbol 12‐myristate 13‐acetate (PMA) into macrophage‐like cells that are competent for innate immune sensing. We used RT normalisation, as described above, to equalise differences in viral production and ensure similar viral dosage between the mutants. We chose this as our preferred quantification as this detects only active RT in viral particles and was close to both p24 and TIRF production measurements. Infection resulted in the stimulation of CXCL10 and IFIT1 immune transcripts, with K158A provoking a substantially stronger response than WT virus (Fig EV1G). Previous reports have shown that differentiation of THP‐1 cells results in SAMHD1 activation by dephosphorylation, which potently restricts HIV‐1 infection and can decrease the stimulation of innate immune response to HIV particles (Hrecka *et al*, 2011; Cribier *et al*, 2013). To overcome this, we generated a CRISPR‐derived THP‐1 cell line knocked out for the HIV restriction factor SAMHD1 (herein named THP‐1/SAMHD1 KO cells; Fig EV1H). THP‐1/SAMHD1 KO cells remained fully competent for innate immune sensing and produced proteins from interferon‐stimulated genes (ISGs) such as CXCL10 and IFIT1 after exposure to LPS (Fig 1F). This is in line with previous data showing that SAMHD1‐silenced THP‐1 were able to respond to a range of stimuli, including transfection of herring testis DNA (HT‐DNA) or exposure to 2′3′‐cGAMP (Sumner *et al*, 2020). As expected, SAMHD1 knockout increased HIV‐1 infection compared with THP‐1 wild‐type cells (Fig 1G). PMA‐treated THP‐1/SAMHD1 KO cells were therefore used in viral challenge experiments where immune detection was monitored by measuring the transcription of immune genes CXCL10, IFIT1 and IFIT2. Infection of THP‐1/SAMHD1 KO cells with K158A resulted in a > 10‐fold higher expression of each immune gene compared with WT (Fig 1H). The addition of the second‐site mutation T8I significantly reduced immune gene transcription, although not to the levels of WT virus or T8I alone (Fig 1H). To confirm these results, we also examined the secretion of cytokines CXCL10 and TNFα. We observed significantly more CXCL10 and TNFα secretion following infection with K158A compared with WT (Fig 1I and J). Meanwhile, the double mutant K158A/T8I showed reduced cytokine secretion close to WT levels. These results are consistent with K158A being more easily sensed by host cells during infection and the addition of T8I partially restoring HIV‐1 immune evasion.

To investigate the nature of the immune activation induced by K158A in more detail we examined a wider panel of immune transcripts. This revealed that a broad spectrum of cytokines and chemokines are upregulated during K158A infection, including several Interferon (IFN) stimulated genes (Fig 1K). Once again, the introduction of the capsid stabilising mutation T8I onto the K158A background (K158A/T8I) reversed immune induction (Fig 1K). We hypothesised that the induction of cytokines by IP6‐deficient K158A virus during infection may be sufficiently strong to induce a paracrine antiviral response. To test this, we performed a media conditioning experiment in which the medium from cells infected with different viruses was transferred onto fresh THP‐1/SAMHD1 KO cells. WT HIV‐1 was then added to the fresh cells 18 h post media transfer and infection monitored by GFP expression. In the case of a strong immune response, cytokines present in the media would be predicted to reduce subsequent infection (Fig 1L). Consistent with this prediction, the infectivity of WT virus was decreased in cells treated with K158A‐conditioned media but not with WT or K158A/T8I‐conditioned media confirming that K158A virus induced immune response (Fig 1M). Taken together, the data show that HIV‐1 mutant K158A is sensed by host cells during infection, resulting in sufficient cytokine and chemokine upregulation to induce a paracrine antiviral state. The ability of T8I to substantially reverse immune sensing is consistent with the notion that IP6 is required to produce mature capsids capable of infecting cells without stimulating a strong immune response.

### Capsid‐sensing is not responsible for detection of IP6‐deficient HIV particles

**Figure 2:**
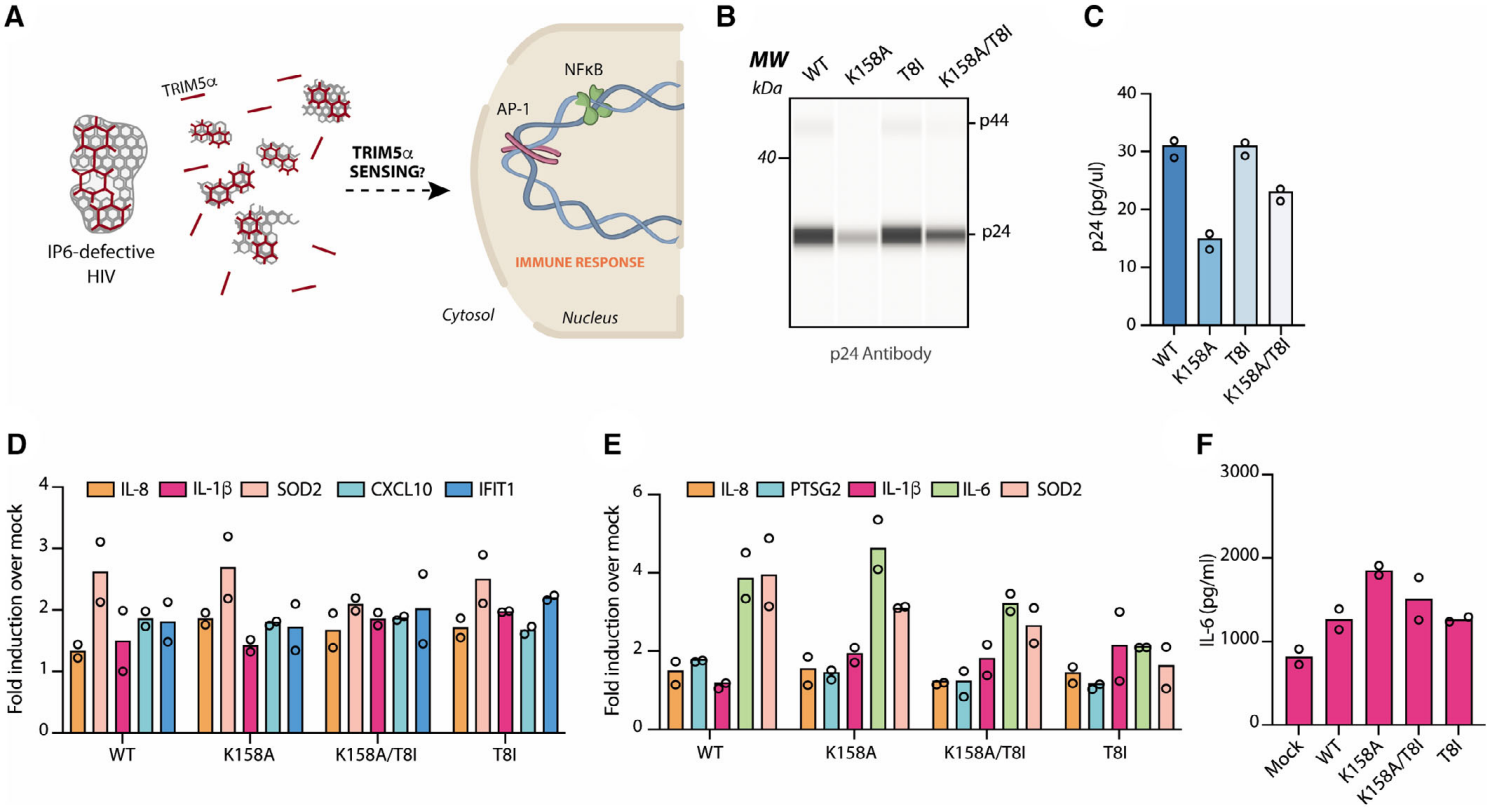
Capsid‐sensing is not responsible for the detection of IP6‐deficient HIV particles A
Schematic of possible capsid‐sensing mechanism of IP6‐deficient HIV virions by the capsid sensor TRIM5α.B
p24 quantification by capillary western blot of the empty VLPs produced with the indicated capsid mutants.C
Quantification of p24 blot shown in (B).D
qPCR quantification of the indicated chemokine transcripts from PMA‐treated THP‐1/SAMHD1 KO cells transduced for 24 h with the indicated VLPs mutants.E
qPCR quantification of the indicated chemokine transcripts level from PMA‐treated THP‐1/SAMHD1 KO cells transduced for 24 h with 50 ng/RT of the indicated HIV‐1 GFP capsid mutants.F
Protein quantification of IL‐6 by Luminex™ from PMA‐treated THP‐1/SAMHD1 KO cells infected with the indicated HIV‐1 mutants. Data information: All mean data represent *n* = 2 biological replicates.

The presence of incomplete capsid lattice structures in IP6‐deficient K158A virions (Fig 1A) suggested that one way the mutant may provoke host sensing is through detection by a capsid sensor such as the retroviral capsid sensor TRIM5α (Pertel *et al*, 2011; Ganser‐Pornillos & Pornillos, 2019). TRIM5α undergoes templated assembly into a hexameric lattice in the presence of a capsid hexameric lattice, leading to the activation of its RING E3 ligase domains. This in turn results in the synthesis of K63 ubiquitin chains and the activation of immune signalling pathways (Pertel *et al*, 2011; Fletcher *et al*, 2018; Fig 2A). To test whether the detection of K158A is through its capsid lattice structures, we produced empty HIV virus‐like particles (VLPs) that lack a viral RNA genome (Fig 2B). As expected, K158A VLPs were produced at lower levels compared with the other capsid mutants while the addition of T8I mutation (K158A/T8I) mostly rescued VLP production (Fig 2B and C). Challenge of THP‐1‐SAMHD1 KOs with the mutant empty VLPs was then carried out after equalising VLPs by p24 capillary western blot. Previous work has shown that capsid detection by TRIM5α induces the pro‐inflammatory cytokines IL‐8, IL1β and SOD2 (preprint: Zuliani Alvarez *et al*, 2022). However, we observed no increased upregulation of these cytokines upon challenge with K158A VLPs compared with WT (Fig 2D). Of note, CXCL10 or IFIT1 that was upregulated during infection with HIV virions (Fig 1H), was not stimulated during empty VLP infection (Fig 2D). To confirm this result, we repeated the experiment with genome‐containing particles and similarly observed no difference in the transcription of IL‐8, IL1β and SOD2 (Fig 2E) or IL‐6 secretion (Fig 2F) between K158A and WT. Taken together, this suggests that the increased immune detection of K158A is not based on previously described capsid‐sensing.

### Sensing of IP6‐deficient HIV‐1 capsid is dependent on postentry synthesis of viral DNA

**Figure 3:**
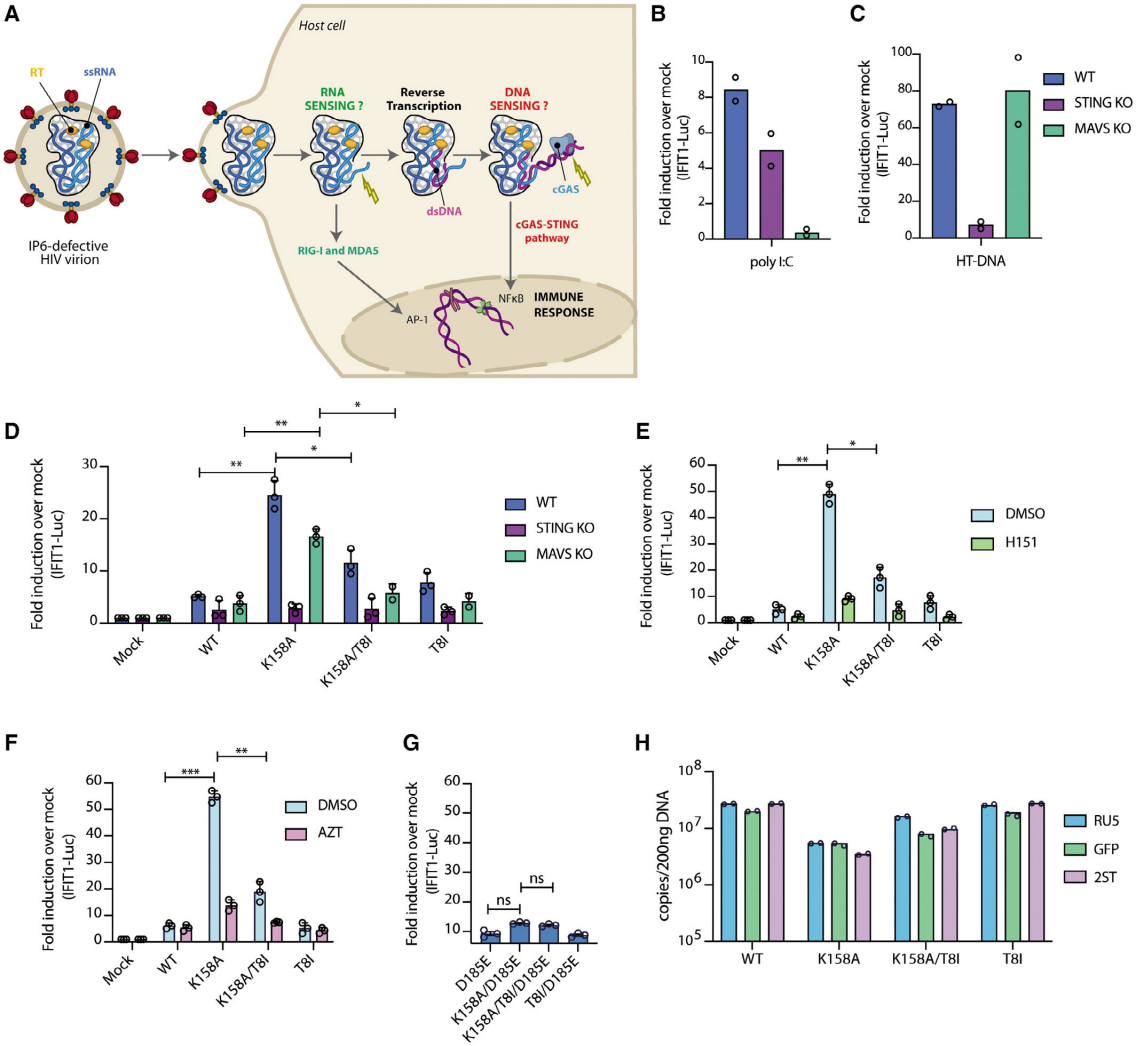
Sensing of IP6‐deficient HIV‐1 capsid is dependent on the postentry synthesis of viral double‐stranded DNA A
Schematic of ssRNA and dsDNA sensing mechanism of IP6‐deficient HIV virions.B–D
IFIT1‐Luc reporter activity from PMA‐treated THP‐1/SAMHD1 KO cells stimulated by transfection with either poly I:C (0.5 μg/ml) (B) or HT‐DNA (0.1 μg/ml) (C) or infected for 24 h with 50 ng/RT of the indicated HIV‐1 GFP capsid mutants (D). (B) and (C) mean data represent *n* = 2 biological replicates.E, F
IFIT1‐Luc reporter activity from PMA‐treated THP‐1/SAMHD1 KO infected for 24 h with 50 ng/RT of the indicated HIV‐1 GFP capsid mutants in the presence of DMSO or 0.5 μg/ml H151 (E) or AZT (1 μM) (F).G
IFIT1 activity quantification from THP‐1/SAMHD1 KO cells infected with 50 ng/RT of the indicated HIV‐1 capsid mutants carrying a D185E mutation in reverse transcriptase.H
Quantification of the RU5 (early), GFP (middle) and 2ST (late) transcripts by qPCR from PMA‐treated THP‐1/SAMHD1 KO cells transduced for 6 h with the indicated HIV‐1 capsid mutants. Mean data represent *n* = 2 technical replicates. Data information: Error bars depict the mean ± SEM. Unless otherwise indicated, all mean data represent *n* = 3 biological replicates. Statistical analyses were performed with the Student's *t* test. *P < 0.05, **P < 0.01, ***P < 0.001, *n.s*. = not significant.

As the detection of defective capsids did not appear to be the basis of the increased sensing of IP6‐deficient K158A virions, we investigated whether detection involves nucleic acid sensing (Fig 3A). To do so, we took advantage of THP‐1 cell lines stably expressing luciferase under the control of the IFIT1 promoter (herein named IFIT1‐luc THP‐1 cells) and which have been edited using CRISPR/Cas9 technology to delete genes encoding the DNA sensing adaptor STING or the RNA sensing adaptor MAVS (Sumner *et al*, 2020). As previously shown, IFIT1‐luc THP‐1‐STING KO cells did not respond to transfected herring testis (HT) DNA but ISG induction was detected in response to the RNA mimic poly I:C (Fig 3B and C). Conversely, IFIT1‐luc THP‐1‐MAVS KO cells were activated by HT‐DNA but not poly I:C (Fig 3B and C; Sumner *et al*, 2020). Induction of luciferase in PMA‐treated IFIT1‐luc THP‐1 cells was detected upon infection with K158A mutant and strongly reduced in the double mutant K158A/T8I mutation, further corroborating the results obtained evaluating cytokine and chemokine transcripts. Notably, IFIT1‐Luc induction was almost absent in STING knockout cells but present in the MAVS knockouts, suggesting that synthesised viral cDNA is the predominant viral Pathogen‐Associated Molecular Patterns (PAMP) detected during sensing of K158A (Fig 3D). There was some reduction in the amplitude of IFIT1‐luc induction in MAVS KO cells compared with IFIT1‐luc THP‐1 cells, suggesting there may be a small contribution of HIV‐1 RNA sensing to inflammatory cytokine production upon infection.

To corroborate the results obtained with the CRISPR KO reporter cell lines we repeated the viral challenge experiments in the presence of the STING inhibitor H151 (Haag *et al*, 2018). Use of the inhibitor phenocopied STING knockout and lead to a reduction in IFIT1‐Luciferase expression induced by K158A, confirming that the PAMP‐driving sensing is DNA (Fig 3E). To determine whether this is newly synthesised viral DNA, we compared the ability of capsid mutants to induce an ISG response in the presence or absence of nucleotide‐based reverse transcription inhibitor AZT. IFIT1‐Luc induction by K158A was inhibited by AZT (Fig 3F), supporting a mechanism whereby cells sense viral dsDNA that is exposed in IP6‐deficient capsids. To further confirm this mechanism, we introduced the RT active‐site mutant D185E into each virus, which effectively abolished immune sensing (Fig 3G). Either in the case of AZT or H151 addition or use of the D185E mutant, the addition of the capsid stabilising mutation T8I onto the K158A background (K158A/T8I) strongly decreased IFIT1‐Luc induction (Fig 3E–G).

**Figure EV2:**
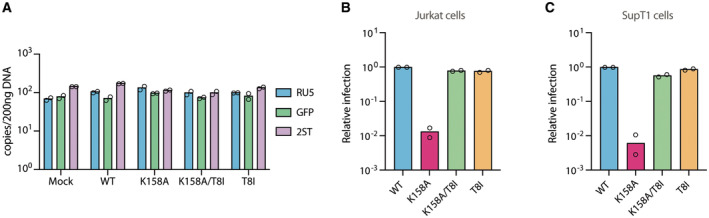
Infection of T‐cell lines A
Quantification of the RU5 (early), GFP (middle) and 2ST (late) transcripts by qPCR from PMA‐treated THP‐1/SAMHD1 KO cells transduced for 6 h with the indicated HIV‐1 capsid mutants boiled for 10 min. Mean data represent *n* = 2 technical replicates.B, C
Relative infection of Jurkat (B) and SupT1 (C) cells infected with 50 ng/RT with the indicated HIV‐1 capsid mutants. Infection is quantified as GFP Area/Phase Area normalised to WT virus. Data information: All mean data represent *n* = 2 biological replicates. Source data are available online for this figure.

Next, we sought to investigate how the instability of the K158A capsid affects the reverse transcription process. We infected cells with our panel of viruses and quantified the level of newly synthesised viral DNA after 6 h using primers specific to minus strand strong‐stop (RU5), first‐strand transfer (GFP) and second‐strand transfer (2ST), which reflects different stages of HIV genome retrotranscription. While the K158A mutant was able to retrotranscribe its genome, this was significantly less efficient than WT virus (Fig 3H). Addition of second‐site mutant T8I partially rescued transcript levels. Both the reduction in DNA synthesis by K158A and rescue by T8I correlate with the relative infectivity of these mutants (Fig 1E). To determine the level of background contamination, for instance from plasmid DNA, the levels of the RU5, GFP and ST transcripts were also quantified in cells infected with boiled‐inactivated virus (Fig EV2B). These results are also in agreement with the TIRF stability data (Fig 1C and D) and suggest that K158A capsids begin to synthesise DNA but collapse before the virus can undergo productive integration. Notably, K158A triggers a stronger immune response in THP‐1‐infected cells despite having lower detectable DNA than WT. This highlights the importance of capsid protection and suggests that DNA protection is more important than DNA production to avoid triggering immune detection during infection.

**Figure 4:**
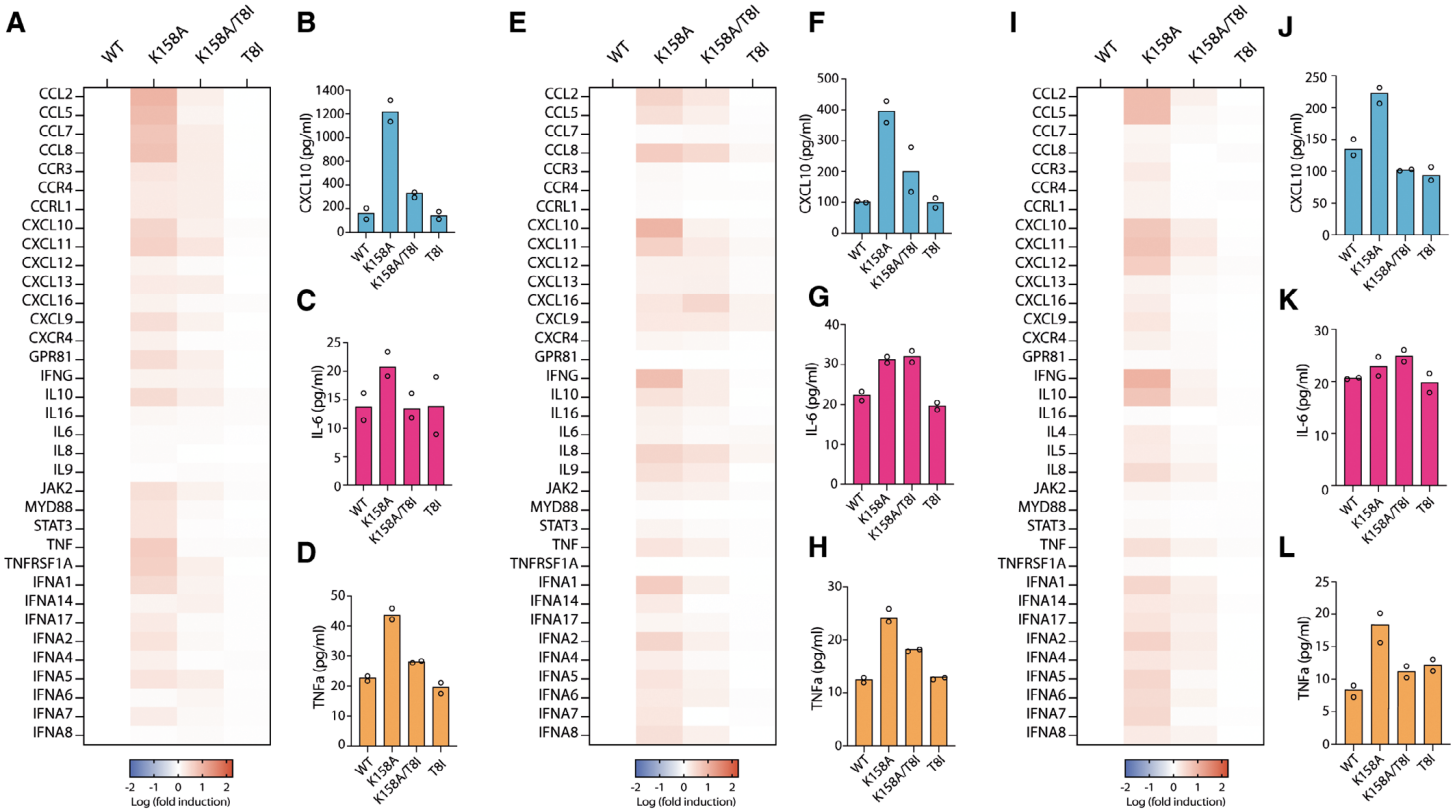
IP6‐deficient particles are sensed in T‐cell lines and human monocyte‐derived macrophages A
TaqMan® Gene Expression Assay qPCR of a panel of human chemokines from human monocyte‐derived macrophages (HMDMs) infected for 24 h with 50 ng/RT of the indicated HIV‐1 mutants. Data are shown as Log fold induction over WT HIV‐1. Mean of *n* = 2 biological replicates experiments are shown.B–D
Protein quantification of CXCL10 (B), IL‐6 (C) and TNFa (D) by Luminex™ from HMDMs infected with the indicated HIV‐1 mutants.E
qPCR of a panel of human chemokines from SupT1 cells infected as in (A).F–H
Protein quantification from SupT1 cells as in (B–D).I
qPCR of the indicated human chemokines from Jurkat T cells infected as in (A).J–L
Protein quantification from infected Jurkat T cells as in (B–D). Data information: All mean data represent *n* = 2 biological replicates.

### IP6‐deficient particles are sensed in T‐cell lines and primary monocyte‐derived macrophages

Having observed that the host innate immune system can sense capsids that form in IP6‐deficient virions, we wondered whether this also occurs in primary macrophages and T‐cell lines. We therefore isolated PBMCs from peripheral blood and generated Monocyte Derived Macrophages (MDMs). Infection of MDMs with the IP6‐deficient mutant K158A generated a strong inflammatory response both at the transcript and secreted protein level, with the exception of the TRIM5α‐associated cytokine IL‐6 (Fig 4A–D). The other capsid mutants, including the double mutant K158A/T8I, induced very little response. These results are consistent with the transcriptional profile observed in PMA‐treated and infected THP‐1 cells. HIV‐1 replicates efficiently in primary T cells *in vitro* but only after they are activated, typically by cross‐linking the T‐cell receptor to mimic antigen stimulation (Maier *et al*, 2000; Wilen *et al*, 2012). Therefore, to test whether T cells are also able to sense IP6‐deficient capsids, we evaluated the immune profile of Jurkat and SupT1 cells, which are widely used models of T‐cell infection (Jordan *et al*, 2003). Infection experiments in such cell lines showed the same phenotype observed in the case of HEK293T cells with a 2‐log defect in K158A infectivity compared with HIV‐WT (Fig EV2B and C). Note that the same high viral dose was used as in the corresponding sensing experiments and under these conditions the mild defect in T8I compared with WT, as reported in some studies (Mallery *et al*, 2021), is not observed. Just as with THP‐1s and primary human macrophages, the strongest cytokine response in both cell lines was observed upon infection with the K158A mutant, particularly CXCLs, CCLs and IFNγ (Fig 4E and I). Furthermore, CXCL10 and TNFα secreted protein levels were higher during K158A infection compared with the other capsid mutants, again excepting TRIM5α‐associated cytokine IL‐6 (Fig 4F–H and J–L). Interestingly, the amplitude of the inflammatory response both at the transcript and protein level differs among the two T‐cell lines, with a higher stimulation of SupT1s compared with Jurkats. Together, these data suggest that infection by viruses with capsids assembled in the absence of enriched IP6 (K158A) induces an innate immune response in macrophages and T‐cell lines that is reversed by the addition of a second‐site mutation that restores IP6‐packaging (K158A/T8I).

Ever since early pioneering studies on capsid stability (Forshey *et al*, 2002), there has been a gradual reassessment of the importance of maintaining an intact HIV‐1 capsid postentry. It is now clear that rather than collapsing immediately after fusion, the capsid remains intact for many hours during its transit through the cell: Ground‐breaking high‐content microscopy approaches, including correlative light and electron microscopy (CLEM) and cryoET, have revealed that intact capsids can pass through the nuclear pore (Li *et al*, 2021; Zila *et al*, 2021). Imaging these capsids and their contents has demonstrated that uncoating and release of viral cDNA happens inside the nucleus, only minutes before integration (Burdick *et al*, 2020; Müller *et al*, 2021). This realisation of capsids' true longevity has been accompanied by the discovery of new host factor binding sites on capsid in addition to the CypA binding loop (Luban *et al*, 1993), including the CPSF6/Nup153/PF74 interface (Lee *et al*, 2010; Matreyek & Engelman, 2011; Price *et al*, 2012, 2014) and the R18 charged pore (Jacques *et al*, 2016). Capsid must remain assembled to preserve these interfaces, providing functional evidence consistent with imaging data. For instance, the binding site for CPSF6 and Nup153, both factors that only engage once HIV‐1 reaches the nucleus, is located at the interface between monomers in capsid assemblies (Price *et al*, 2014). Maintaining a stable capsid is important not only for delivering the viral genome into the nucleus but also for preventing it from being sensed by host immunity. Indeed, the capsid of HIV‐1 has evolved a dNTP import system that allows it to fuel DNA synthesis while maintaining its integrity for as long as possible (Jacques *et al*, 2016). Again, functional and imaging data agree that the final stages of DNA synthesis occur in the nucleus itself, immediately prior to uncoating and integration (Dharan *et al*, 2020; Francis *et al*, 2020; Selyutina *et al*, 2020).

Recent work has identified a new cofactor, the metabolite IP6, as a key player in both assembling and stabilising the capsid (Mallery *et al*, 2018; Dick *et al*, 2018b). Reducing IP6 levels inside budded virions either by reducing metabolite levels in producer cells, or through Gag mutations, disrupts capsid formation as assessed by cryoET (Mallery *et al*, 2021; Renner *et al*, 2021, 2023) and has a drastic impact on both viral production and particle infectivity (Mallery *et al*, 2019, 2021; Ricana *et al*, 2020; Sowd *et al*, 2021). This is supported by *in vitro* assembly data, where IP6 is required for mature conical capsids to form (Dick *et al*, 2018b; Mallery *et al*, 2021; Renner *et al*, 2023). *In vitro* data also suggests that IP6 is required once capsids have formed in order to maintain their stability. Single‐molecule TIRF experiments have shown that preformed capsids collapse within minutes, whereas in the presence of exogenous IP6, they are stable for many hours (Mallery *et al*, 2018, 2019; Márquez *et al*, 2018; Renner *et al*, 2021). Exogenous IP6 is also required to stabilise capsids during encapsidated reverse transcription (ERT) (Mallery *et al*, 2018, 2019; Jennings *et al*, 2020; Sowd *et al*, 2021), consistent with the notion that the accumulation of DNA inside the capsid provides an uncoating force. While both TIRF and ERT data support a role for IP6 after the capsid has formed, depletion of IP6 from target cells through the knockout of inositol phosphate kinases has not been demonstrated to alter the infectivity of wild‐type HIV‐1. However, this is likely because the affinity of the capsid for IP6 is extremely high (< nM), whereas even upon knockout of IPPK or IPMK there are > nM levels of IP5 or IP5 & 6, respectively (Mallery *et al*, 2019; Renner *et al*, 2023).

The data presented here suggest that IP6 is also critical in facilitating HIV‐1 immune evasion. HIV‐1 with a Gag mutation (K158A) that prevents IP6 from being enriched into viral particles induces a robust innate immune response upon infection. Sensing occurs in a variety of cell types including THP‐1s, primary human macrophages and T‐cell lines, and results in the upregulation of a panel of cytokines and chemokines. These secreted cytokines are sufficient to induce a paracrine antiviral state and reduce HIV‐1 infection. We further show that immune evasion can be restored to a K158A mutant virus by complementing it with the second‐site mutation T8I. T8I stabilises the 6HB in immature hexamers that comprise the immature lattice in budding HIV‐1 virions (Mallery *et al*, 2021). T8I also restores the ability of K158A to package IP6, rescues mature capsid formation and infection (Mallery *et al*, 2021). The data here reveals that T8I also reduces the sensing of K158A by host innate immunity.

Disrupting IP6 enrichment into HIV‐1 virions has two potential consequences. First, it can disrupt the capsid assembly process, leading to virions that contain aberrant lattice structures and fewer single capsid cores (Fig 1A and B). Second, any capsids that do form will lack IP6 to stabilise them and are likely to rapidly collapse when no longer enclosed in the viral membrane. Even if there is IP6 available in target cells, there are hundreds of binding sites per capsid and collapse may occur before reaching IP6:capsid equilibrium. As capsid mutant K158A displays both defective assembly and stability, the immune sensing it triggers upon infection could be caused by either. Lattice structures can be sensed by TRIM5α, which undergoes templated assembly on hexameric lattices into an active form that synthesises K63 ubiquitin chains, an immune second messenger (Pertel *et al*, 2011; Fletcher *et al*, 2015). However, the immune activation we observed upon infection with K158A did not have the cytokine signature associated with TRIM5α. Furthermore, when we infected cells with VLPs that contain capsid protein but lack viral RNA, K158A was no longer sensed. Together, this suggests that the immune response to K158A virus is not caused by the direct detection of improperly assembled capsids. Given the requirement for viral nucleic acid, this indicates that what is being sensed is either genomic RNA or double‐stranded DNA (dsDNA) that is synthesised during reverse transcription. Also, the heterogeneity of K158A virions and the presence of particles, which may contain multiple viral genomes could contribute to the sensing of either nucleic acid. Knockout of the RNA sensing adaptor MAVS had a limited effect on immune activation, while STING knockout significantly reduced it suggesting DNA is the primary PAMP. Use of a STING antagonist further supported this conclusion, while AZT inhibition of RT showed that it is newly synthesised viral DNA that is sensed. Importantly, fewer early, middle and late transcripts were detected during K158A infection suggesting it is the accessibility of DNA rather than abundance of viral DNA that determines sensing. Previously, we have reported that IP6 is required to stabilise the capsid during encapsidated DNA synthesis (Mallery *et al*, 2018). Consistent with these data, the K158A virions used here were intrinsically less stable than WT, with immune evasion and stability being concomitantly restored by second‐site mutant T8I. Taken together, this suggests that sensing is caused by incomplete K158A capsids entering cells and reverse transcribing or complete capsids that undergo partial DNA synthesis and then collapse before reaching the nucleus.

The results presented here support previous observations highlighting the importance of the capsid in protecting HIV‐1 from innate sensing (Rasaiyaah *et al*, 2013; Sumner *et al*, 2020) and reveal how this capsid protection is dependent upon IP6. Our study is also in line with a previous report (Sumner *et al*, 2020) showing that when Gag processing is perturbed by protease inhibition this leads to increased sensing. Where exactly unstable capsids lose integrity and are sensed remains to be determined. Given that HIV‐2 cGAS sensing has been reported in the nucleus (Lahaye *et al*, 2018), one possible approach to address this may be to determine whether sensing is driven by cGAS localised in the cytoplasm or nucleus. Recently we showed that HIV‐1 can easily become independent of IP6 for immature lattice assembly and viral production with just two mutations (Renner *et al*, 2023). However, when HIV‐1 fails to enrich IP6 into virions then it cannot efficiently assemble mature capsids and is poorly infectious. While this demonstrates that IP6 is required for mature capsid assembly in virions, why HIV‐1 has evolved a capsid strategy that makes it so dependent upon IP6 remains an open question. IP6 promotes assembly by coordinating charged pores in mature capsomers. However, as this is not the only way to build and stabilise a viral capsid, we speculate IP6 is needed because of the charged pores, rather than vice versa. The positively charged pores in HIV‐1 capsids are ideal for importing dNTPs, suggesting this may be why they have evolved, but they create charge repulsion that needs to be neutralised by a negatively charged molecule like IP6. In any case, identifying the importance of IP6 in allowing HIV‐1 to evade host immunity suggests that inhibiting this interaction could be an effective approach to prevent HIV‐1 infection and promote host immunity.

## Materials and Methods

### Cells and plasmids

Human embryonic kidney (HEK) 293T CRL‐3216 cells were purchased from American Type Culture Collection. All cells are mycoplasma free and they are regularly tested. HEK293T was cultured in Dulbecco's modified Eagle's medium with 10% foetal bovine serum (FBS), 2 mM L‐glutamine, penicillin (100 U/ml) and streptomycin (100 mg/ml; Gibco) at 37°C with 5% CO_2_.

THP‐1 cells were maintained in RPMI (Gibco) supplemented with 10% FBS and Pen/Strep. THP‐1‐IFIT1 cells that had been modified to express *Gaussia* luciferase under the control of the IFIT1 promoter were described previously (Mankan *et al*, 2014). THP‐1 Dual Control and STING KO and MAVS KO cells were previously described (Sumner *et al*, 2020). Jurkat and SupT1 T‐cell lines and PBMCs were maintained in RPMI 1640 with L‐glutamine (Corning) and supplemented with 10% FBS (GenClone), penicillin (100 U/ml) and streptomycin (100 mg/ml). STING inhibitor H151 was obtained from Invitrogen.

Mutant construct D185E was generated with the New England Biolabs (NEB) Q5 site‐directed mutagenesis kit (NEB, E0554) against pCRV GagPol WT and mutants (K158A, K158A/T8I, T8I; Mallery *et al*, 2021) using primers designed using the NEBaseChanger online tool.

### Generation of THP‐1‐SAMHD1 KO cells

THP‐1 Cas9 cells, generated with lentiCAS9‐Blast (Addgene #52962), were a kind gift from Andres Floto (University of Cambridge). To generate SAMHD1 CRISPR knockout cell population we used Target Guide Sequence Cloning Protocol (Joung *et al*, 2017).

Guides were designed with CHOPCHOP online tool (Labun *et al*, 2019) and cloned into lentiGuide‐Puro (Addgene #52963) vector. Lentiviruses were generated in HEK293T cells and used to transduce THP‐1 Cas9 cells. Shortly, cells were pelleted and resuspended in antibiotic‐free RPMI. 10^6^ of cells were transduced with 300 μl of lentivirus‐containing supernatant in the presence of polybrene (10 μg/ml, sc‐134220) by spinfection for 30 min at 800 *g*. For the control population, cells were transduced with 300 μl of empty guide vector. For the KO, a mix of 100 μl of each guide supernatant was used (3 × 100 μl). After 48 h 1 μg/ml of puromycin was added to select transduced cells. To check for KO efficiency cells were differentiated with PMA, lysed with Triton X‐100 and processed for WB to detect SAMHD1 (rabbit Proteintech 12586‐1‐AP) and Actin.

CRISPR Guides:

1. 5′ CACCGGTCATCGCAACGGGGACGCT; 3′ CCAGTAGCGTTGCCCCTGCGACAAA
2. 5′ CACCGGTGTATCAATGATTCGGACG; 3′ CCACATAGTTACTAAGCCTGCCAAA
3. 5′ CACCGCGTGGATTTGAACCAATCGC; 3′ CGCACCTAAACTTGGTTAGCGCAAA

### Virus production

VSV HIV‐1 pseudotypes were produced as previously described (Mallery *et al*, 2021). Briefly, 2.5 × 10^6^ cells were plated in a 10‐cm dish the day before. Transfection mixtures were made using 200 μl of Opti‐MEM (Gibco), 1 μg of pMDG2, 1.5 μg of pCSGW, 1 μg of pCRV GagPol mutant plasmids and 12 μl of FuGENE6 (Promega). Mixtures were incubated at room temperature for 15 min and then added to 10‐cm dishes. Viral supernatants were harvested 48 h after transfection, filtered through a 0.45‐μm filter and stored at −80°C.

For tomography, the virus was produced in HEK293T as described above. Supernatants were harvested and passed through a 0.45‐μm filter, followed by a 0.22‐μm filter. The particles were concentrated by ultracentrifugation over a 20% (w/v) sucrose cushion (2 h at 28,000 rpm in a Beckman SW32 rotor, Beckman Coulter Life Sciences). Resuspended particles were applied to a 6–18% iodixanol gradient (1.2% increment steps) and centrifuged for 1.5 h at 250,000 *g* in a Beckman SW40 rotor (Beckman Coulter Life Sciences) (34). The virus‐containing fraction was diluted in 1:10 phosphate‐buffered saline (PBS) and concentrated by ultracentrifugation (45 min at 38,500 rpm in a Beckman SW40 rotor, Beckman Coulter Life Sciences). The pellet was resuspended in PBS and incubated at 4°C overnight to allow full resuspension.

### Virus quantification

The level of RT enzyme was quantified using quantitative reverse transcription polymerase chain reaction (PCR). Briefly, 5 μl of viral supernatant was mixed with 5 μl of lysis buffer [0.25% Triton X‐100, 50 mM KCl, 100 mM tris–HCl (pH 7.4) and 40% glycerol] and 0.1 μl of ribonuclease (RNase) inhibitor and incubated for 10 min at room temperature before diluting to 100 μl with nuclease‐free water. Two microliters of lysate were added to 5 μl of TaqMan Fast Universal PCR Mix, 0.1 μl of MS2 RNA, 0.05 μl of RNase inhibitor and 0.5 μl of MS2 primer mix, to a final volume of 10 μl. The reaction was run on an ABI StepOnePlus Real‐Time PCR System (Life Technologies), with the additional reverse transcription step (42°C for 20 min).

### Capsid quantification and stability by TIRF imaging

Cell culture supernatants containing lentiviruses were used for the quantification and stability of VLPs. 8‐well glass bottom Ibidi‐dishes were first covered with Poly‐L‐lysine (Sigma P4707) for 1 h and then washed with water and PBS. Viruses were added to wells for 1 h binding at the same dilution for quantification (1 in 6). For stability experiments, K158A virus was undiluted to obtain more virus particles for analysis. After wash, samples were fixed with 4% formaldehyde (Thermo Scientific 28908) for 20 min and permeabilised with 0.1% Triton X‐100 for 5 min. Capsids were labelled with P24/25 antibody (mix of Mab EF7 and 38‐96k), followed by secondary Alexa Fluor 488 against mouse IgG1.

For Streptolysin‐O experiments (SLO, Sigma, S5265) we diluted it with 50 μl PBS and 2 mM TCEP to obtain ~ 5 μM stock solution. Viruses bound to dishes were treated with 50 nM SLO in presence of 50 μM IP6 (Sigma, 593648) for 30 min at 37°C in a buffer containing 100 mM Tris–pH 8.0 and 100 mM NaCl. After wash with the buffer, samples were fixed and processed as above. For control condition, the viruses were fixed straight after the binding step.

All images were acquired using a Nikon TIRF inverted microscope with a 100×/1.49NA oil‐immersion objective, a 1.5× intermediate magnification and Prime95B sCMOS camera from Photometrics resulting in a 74 nm pixel size. All laser powers were kept identical during the imaging. The analysis of the images was performed in Fiji where images were median filtered and background subtracted. An intensity threshold was used to create a mask and a watershed step allowed separating the touching particles. The threshold was adjusted to select particles of interest: 100 for quantification and 400 for stability test. Finally, ROI was filtered by area within 5–500 pixels and mean fluorescence intensities measured in the original image. Graphs were made using GraphPad Prism.

### Infection assays

For infection experiments, THP‐1, SupT1, Jurkat and PBMCs cells were seeded at 5 × 10^4^ cells per well into 96‐well plates and infected 16 h after plating. 50 ng/RT of HIV‐1 was added to each well for every condition, and the plates were left for 24 h before harvesting for qPCR and 48 h for cytokine protein quantification.

THP‐1 cells were infected at a density of 5 × 10^5^ cells/ml. For differentiation, THP‐1 cells were treated with 50 ng/ml phorbol 12‐myristate 13‐acetate (PMA, PeproTech) for 48 h. Luciferase reporter assays and qPCR were performed in 96‐well plates and ELISA in 24‐well plates. Infection levels were assessed at 48 h postinfection through the enumeration of GFP‐positive cells using Incucyte. Infections were performed in the presence of polybrene (8 μg/ml, Sigma). The input dose of virus was normalised either by RT activity or genome copies (measured by qPCR) in the case of D185E.

### Luciferase reporter assays

*Gaussia* luciferase activity was measured by transferring 10 μl supernatant to a 96‐well assay plate and mixing 50 μl per well of coelenterazine substrate (Nanolight Technologies). Luminescence was analysed using a PHERAstar FSX Microplate Reader (BMG‐Labtech). Data were normalised to a mock‐treated sample to generate a fold induction.

### ISG–qPCR

RT–qPCR was performed using the Cell‐to‐CT Kit (Invitrogen). Briefly, plates were immediately frozen at −70°C 24 h after infection. Next, plates were thawed at 4°C and 1 volume of lysis buffer (0.25% Triton X‐100), 50 mM KCl, 100 mM Tris–HCl pH 7.4, glycerol 40% and RNAsecure (1/100) with DNaseI 1/100 added to wells and mixed gently by pipetting up and down few times. After 10 min of lysis, cell lysates were transferred to PCR plates and virus inactivated at 95°C for 5 min. 2 μl Stop solution was added to terminate the reaction. 10 μl RT Buffer (2×) was mixed with 0.5 μl RT enzyme mix and 5 μl lysate and incubated at 37°C 1 h, followed by 95°C 5 min run on an ABI StepOnePlus Real‐Time PCR System (Life Technologies) (37°C 1 h, 95°C 5 min). The qPCR was performed on an ABI StepOnePlus Real‐Time PCR System (Life Technologies) using 2 μl RT product, 5 μl TaqMan Fast Universal PCR Mix (ABI) and 0.5 μl GFP primer‐probe (GFP) in a 10 μl reaction. Expression of each gene was normalised to internal control (ActB), and these values were then normalised to mock‐treated control cells to yield a fold induction. The following genes were evaluated: ActB (Hs99999903_m1), CXCL10 (Hs01124251_g1), IFIT1 (Hs03027069_s1), IFIT2 (Hs00533665_m1), PTGS2 (Hs00153133_m1), SOD2 (Hs00167309_m1), IL‐6 (Hs00174131_m1), IL‐1B (Hs00174097_m1), PTGS2 (Hs00153133_m1), IL‐8 (Hs00174103_m1).

The cDNA used for the ISG–qPCR was then used to analyse a panel of chemokine transcripts using TaqMan Array 96‐Well FAST Plate‐Human Chemokines (Cat. 4418729) following the manufacturer's instructions (ThermoFisher).

### Reverse transcription in cells

Cells were seeded the day before at 80% confluence and infected with the different IP6‐deficient mutants with 50 ng/RT the next day. Six hours postinfection, cells were lysed and DNA was extracted using QIAamp DNA Mini Kit (Qiagen). Reverse transcript products were detected using TaqMan Fast Universal PCR Mix (ABI) and RU5 primers to detect strong‐stop DNA (RU5 forward: 5′‐TCTGGCTAACTAGGGAACCCA‐3′; RU5 reverse: 5′‐CTGACTAAAAGGGTCTGAGG‐3′; and RU5 probe 5′‐(FAM) TTAAGCCTCAATAAAGCTTGCCTTGAGTGC(TAMRA)‐3′); GFP primers to detect first‐strand transfer products (GFP Forward (CAACAGCCACAACGTCTATATCAT), GFP Rreverse (ATGTTGTGGCGGATCTTGAAG) and probe GFP (5′‐(FAM) CCGACAAGCAGAAGAACGGCATCAA‐(TAMRA)‐3′) and primers for second‐strand transfer products (2ST forward: 5′‐TTTTAGTCAGTGTGGAAAATCTGTAGC‐3′; 2ST reverse: 5′‐TACTCACCAGTCGCCGCC‐3′; and 2ST probe: 5′‐(FAM) TCGACGCAGGACTCGGCTTGCT(TAMRA)‐3′).

### Cryo‐tomography

Ten‐nanometre‐diameter colloidal gold beads were added 1:1 to the purified HIV‐1 mutants. Four‐microliter sample‐gold suspension was applied to a glow‐discharged C‐Flat 2/2 3C (20 mA for 40 s). The grids were blotted and plunge‐frozen in liquid ethane using an FEI Vitrobot Mark II at 15°C and 100% humidity. Tomography of WT virus was performed on a Tecnai F20 transmission electron microscope (FEI/Thermo Fisher Scientific), equipped with a Falcon III Direct Electron detector, operated at 200 kV and controlled using Serial‐EM (35). Tomographic tilt series were acquired under low‐dose conditions with a tilt range between −40° and +40°, angular increments of 3°, defocus between −3 and −6 μm and at a magnification of 50,000× giving a pixel size of 2.09 Å. Tomography of the mutants was performed on an FEI Titan Krios transmission electron microscope at 300 kV equipped with a Gatan K2 summit direct electron detector and a Gatan Quantum energy filter (Gatan imaging filter) as previously described (Renner *et al*, 2021). Tilt series were acquired between −60° and +60° with increments of 3° using a dose symmetric scheme using Serial‐EM. Images were collected at a magnification of 33,000× with 10 frames per tilt and a total dose of ∼120 e−/Å^2^ across all of the tilts. Frames were aligned in Serial‐EM with a final pixel size of 3.667 Å per pixel in the unbinned image stacks. Reconstruction of tomograms was done using IMOD (4.9). The alignment of 2D projection images of the tilt series was performed using gold beads as fiducial markers, and tomograms were reconstructed by back projection. The margin of error (MOE) for each virion category was calculated as described previously (Mallery *et al*, 2021) using the equation *E*=CI*√(*P*(1−*p*)*n*), based on a confidence level (CI) of 95% and where *n* is the total number of virions classified and *p* is the proportion of a particular classification. The number of virions per category was plotted in Fig EV1A, and the MOE was indicated with error bars.

### Cytokine profile

The cytokine quantification was performed using a Luminex™ MAGPIX™ Instrument System following the manufacturer's instructions. With this technology, the concentration of the following cytokines was quantified using Human ProcartaPlex™ Simplex Kit: CXCL10 (Cat. EPX01A‐10284‐901), IL‐6 (Cat. EPX01A‐10213‐901), TNFα (Cat. EPX01A‐10223‐901).

### Capillary‐based immunoblotting

Viral samples were diluted to 5–20 pg/μl in PBS and 3 μl separated by capillary electrophoresis using a separation module for 12–230 kDa (SM‐W008) on Jess (Simple Western, Protein Simple) according to the manufacturer's instructions. Anti‐HIV p24 (183‐H12‐5C) was obtained from the NIH AIDS Reagent Programme, Division of AIDS, NIAID, NIH and was used at 1:100 dilution to ensure saturation and accurate quantification. Detection was via HRP using anti‐mouse detection module (DM‐002). Results were analysed using Compass software (Protein simple) and relative amounts of Gag cleavage products determined.

### Isolation of primary monocyte‐derived macrophages

Human peripheral blood mononuclear cells (PBMCs) were purified from buffy coats (ethical approval REC 16/LO/0997) from the National Health Service Blood and Transplant (Cat no: NC07 [Buffy Coats 50 ml]) by centrifugation over a Ficoll‐Paque density gradient (GE Healthcare). Monocytes were plated in 6‐well TC dishes and differentiated over 6 days into macrophages in RPMI with 10% FCS and P/S in the presence of 40 ng/ml recombinant human MCSF (R&D systems, cat no: 216‐MC‐025). Macrophages were harvested with a cell scraper after incubation of the cells in ice‐cold PBS with 2% FCS and 5 mM EDTA for 10 min. HMDM cells were seeded at 250,000 cells per well of a 24‐well plate or 50,000 cells per well of a 96‐well plate. Macrophages were then infected with 50 ng/RT of HIV‐1 capsid mutants.

### Statistical analyses

Statistical analyses were performed using GraphPad Prism 7 software (GraphPad). All data are shown as mean ± SEM.

### Biosafety

Experiments with VSV‐G HIV‐1 pseudotypes were conducted in the MRC Laboratory of Molecular Biology at Biosafety level 1. Isolation of human peripheral blood mononuclear cells (PBMCs) was conducted in Biosafety level 2 and approved by the LMB Biological Safety Committee.

## Author contributions

**Guido Papa:** Conceptualization; data curation; formal analysis; validation; investigation; visualization; methodology; writing – original draft; project administration; writing – review and editing. **Anna Albecka:** Formal analysis; validation; investigation; methodology; writing – review and editing. **Donna Mallery:** Data curation; formal analysis; investigation; methodology; writing – review and editing. **Marina Vaysburd:** Resources; data curation; formal analysis; validation; methodology; writing – review and editing. **Nadine Renner:** Data curation; formal analysis; visualization; methodology; writing – review and editing. **Leo C James:** Conceptualization; data curation; formal analysis; supervision; funding acquisition; validation; investigation; writing – original draft; project administration; writing – review and editing.

## Disclosure and competing interests statement

The authors declare that they have no conflict of interest.

## Supporting information



Expanded View Figures PDF

Source Data for Expanded View

PDF+

## Data Availability

No large primary datasets have been generated and deposited.
