# Supplementary material for: IP6‐stabilised HIV capsids evade cGAS/STING‐mediated host immune sensing
Source: EMBO Rep. 2023 Mar 27;24(5):e56275. doi: 10.15252/embr.202256275 (PMC10157305; doi:10.15252/embr.202256275)
Supplement: Supplementary file 1 — Expanded View Figures PDF [file EMBR-24-e56275-s004.pdf]

Expanded View Figures

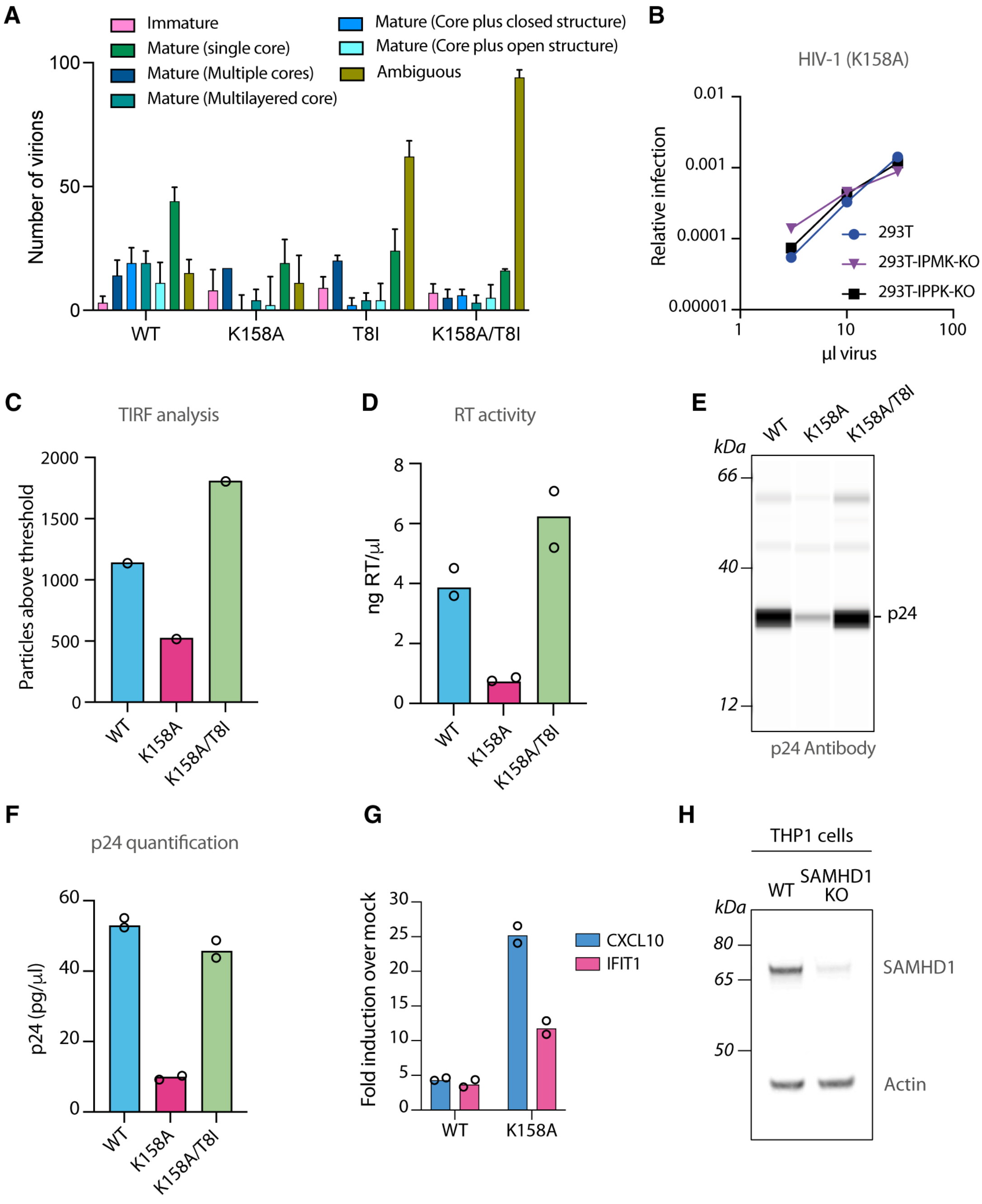

Figure EV1.

 © 2023 The Authors

Figure EV1. Classification of HIV-1 tomograms and quantification and stability of HIV mutants.

A Number of virions per category for each mutant, with error bars showing the margin of error (MOE; see Materials and Methods).
B Titration of virus onto 293T cells with the indicated ng/RT of the HIV-1 capsid mutant K158A expressing GFP. Infection is quantified as GFP Area/Phase Area normalised to Mock.
C Quantification of the indicated HIV mutant virions using TIRF microscopy.
D RT activity quantification of the indicated HIV mutants.
E, F Capillary western blot for p24 of HIV produced with the indicated capsid mutants (E) and p24 quantification (F).
G Quantification of the indicated chemokine transcripts by qPCR from PMA-treated THP-1 WT cells transduced for 24 h with 50 ng/RT of the indicated HIV-1 GFP capsid mutants.
H SAMHD1 western blot of THP-1 WT and SAMHD1 KO cells. Actin was used as a loading control.

Data information: All mean data represent $n = 2$ biological replicates.
Source data are available online for this figure.

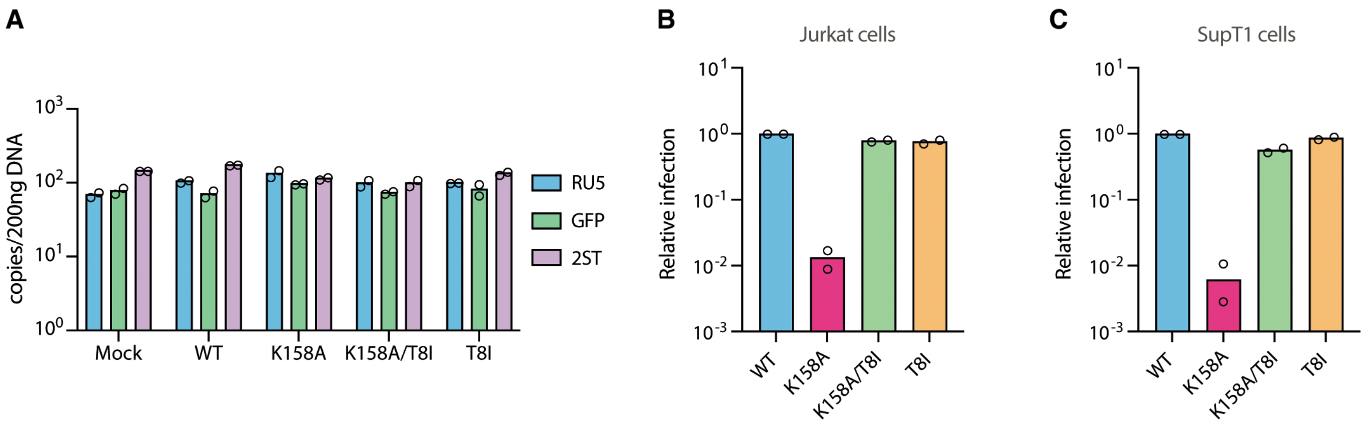

Figure EV2. Infection of T-cell lines.

A Quantification of the RU5 (early), GFP (middle) and 2ST (late) transcripts by qPCR from PMA-treated THP-1/SAMHD1 KO cells transduced for 6 h with the indicated HIV-1 capsid mutants boiled for 10 min. Mean data represent $n = 2$ technical replicates.
B, C Relative infection of Jurkat (B) and SupT1 (C) cells infected with 50 ng/RT with the indicated HIV-1 capsid mutants. Infection is quantified as GFP Area/Phase Area normalised to WT virus.

Data information: All mean data represent $n = 2$ biological replicates.
Source data are available online for this figure.

© 2023 The Authors
