# Supplementary material for: IP6‐stabilised HIV capsids evade cGAS/STING‐mediated host immune sensing
Source: EMBO Rep. 2023 Mar 27;24(5):e56275. doi: 10.15252/embr.202256275 (PMC10157305; doi:10.15252/embr.202256275)

EV1A

	Immature		Mature (Multiple cores)		e (Core plus closed stru		lature (Multilayered core		re (Core plus open struc		Mature (single core)		Ambiguous	
WT	3	2.680068	14	6.286877	19	6.286877	19	4.960826	11	8.363231	44	5.690458	15	5.522437
K158A	8	8.46457	17	0	0	6.20711	4	4.465427	2	11.61244	19	9.640584	11	11.24276
T8I	9	4.526427	20	2.197216	2	3.081966	4	3.081966	4	6.89718	24	8.755306	62	6.419702
K158A/T8I	7	3.709239	5	3.447373	6	2.465627	3	3.159087	5	5.408683	16	0.729159	94	3.159087

ul virus	293T		293T-IPMK-KO		293T-IPPK-KO	
30	0.000685	0.002152	0.000878	0.000874	0.000383	0.001945
10	0.000376	0.000281	0.000358	0.000546	0.000595	0.000254
3	0.000014	9.47E-05	0.000262	0.0000144	0.0000739	0.0000569

EV1C

	WT			K158A			K158A/T8I		
Particle abov	1253	1011	1160	549	586	435	1788	1828	1810

EV1D

	WT			K158A			K158A/T8I		
ng RT/ul	3.655882	4.604688	3.324114	0.718663	0.83978	0.632341	5.319003	7.262917	6.124557

EV1E

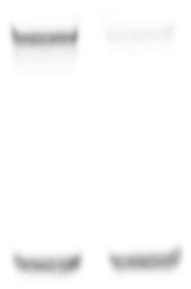

EV1F

p24 quantification

	WT		K158A		K158A/T8I	
p24/ul	54.3	51.6	10.5	9.4366	48.2	43.324

EV1G

Fold induction over Mock

	CXCL10		IFIT1	
WT	4.465531	4.11223	3.14233	4.18837
K158A	23.93654	26.427527	12.726413	10.7634753

EV1H

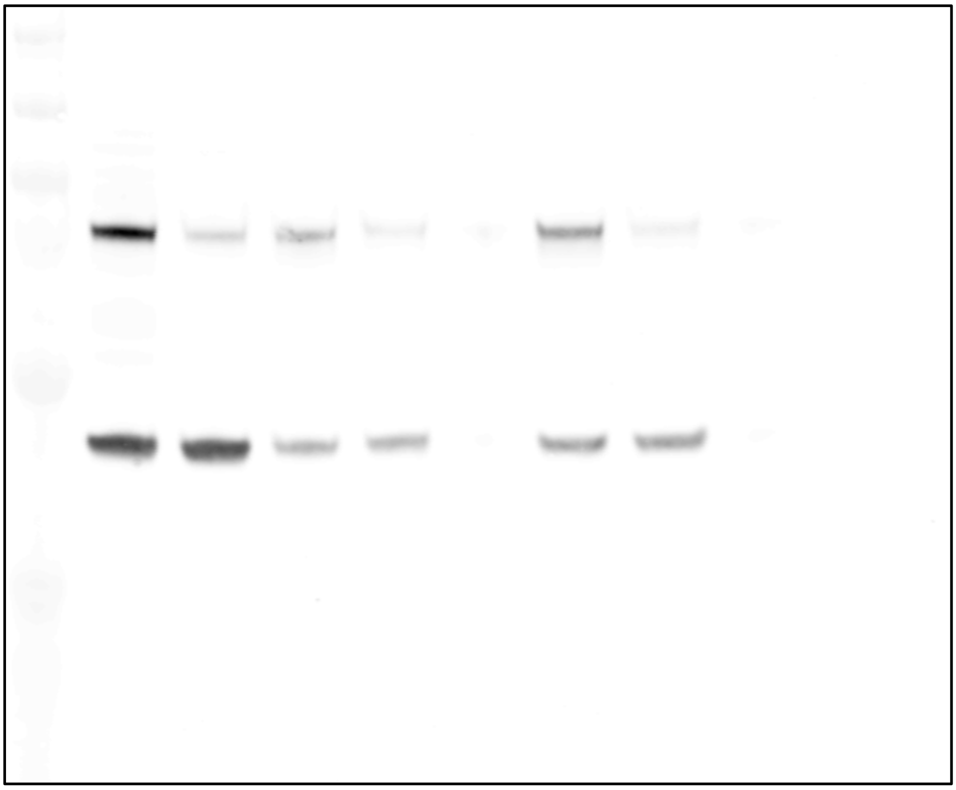

Supplement: Supplementary file 2 — Source Data for Expanded View [file EMBR-24-e56275-s003.zip › EV_Figure_Source_Data/EMBOR-2022-56275V3-Figure_EV1_Source_Data-sd.pdf]
