# Supplementary material for: IP6‐stabilised HIV capsids evade cGAS/STING‐mediated host immune sensing
Source: EMBO Rep. 2023 Mar 27;24(5):e56275. doi: 10.15252/embr.202256275 (PMC10157305; doi:10.15252/embr.202256275)
Supplement: Supplementary file 2 — Source Data for Expanded View [file EMBR-24-e56275-s003.zip › EV_Figure_Source_Data/EMBOR-2022-56275V3-Figure_EV2_Source_Data-sd.pdf]

EV2A

	RU5		GFP		2ST	
Mock	64.51386	74.63326	71.62597	86.38221	148.6217	148.6227
WT	102.3672	110.2379	64.23877	79.38784	180.2883	176.2874
K158A	150.8167	121.8918	99.97163	96.37822	120.7427	112.2673
K158A/T8I	110.8274	90.71765	77.62535	71.62355	90.92784	110.7287
T8I	101.8724	100.7364	67.89385	97.37871	130.248	143.8727

EV2B

Jurkatts

	WT		K158A		K158A/T8I		T8I	
	35.22	39.356	0	0.32104791	0	28.6132563	0	25.0489571
Normalised to WT	1	1	0.017378	0.009116	0.762551	0.812415	0.812341	0.711214

EV2C

SupT1

	WT		K158A		K158A/T8I		T8I	
	41.22	52.14	0	0.10350342	0	25.2500117	0	37.3705054
Normalised to WT	1	1	0.009603	0.002511	0.520907	0.612567	0.82598	0.906611
