# Supplementary material for: IP6‐stabilised HIV capsids evade cGAS/STING‐mediated host immune sensing
Source: EMBO Rep. 2023 Mar 27;24(5):e56275. doi: 10.15252/embr.202256275 (PMC10157305; doi:10.15252/embr.202256275)
Supplement: Supplementary file 4 [file EMBR-24-e56275-s001.pdf]

IP6-stabilised HIV capsids evade cGAS/STING-mediated host immune sensing

Guido Papa, Anna Albecka, Donna Mallery, Marina Vaysburd, Nadine Rennner, and Leo James
DOI: 10.15252/embr.202256275

Corresponding author(s): Leo James (lcj@mrc-lmb.cam.ac.uk)

Review Timeline:

Submission Date:	11th Oct 22
Editorial Decision:	12th Oct 22
Revision Received:	16th Jan 23
Editorial Decision:	15th Feb 23
Revision Received:	28th Feb 23
Editorial Decision:	3rd Mar 23
Revision Received:	6th Mar 23
Accepted:	10th Mar 23

Editor: Achim Breiling

Transaction Report: This manuscript was transferred to EMBO reports following peer review at The EMBO Journal.

(Note: With the exception of the correction of typographical or spelling errors that could be a source of ambiguity, letters and reports are not edited. Depending on transfer agreements, referee reports obtained elsewhere may or may not be included in this compilation. Referee reports are anonymous unless the Referee chooses to sign their reports.)

1st Editorial Decision **12th Oct 2022**

Dear Dr. James,

Thank you for transferring your manuscript to EMBO reports. I now went through your manuscript and the referee reports from The EMBO Journal (attached below). The referees acknowledge that the findings are of interest, but they have raised a number of concerns and suggestions to improve the manuscript, or to strengthen the data and the conclusions drawn.

EMBO reports emphasizes novel functional over detailed mechanistic insight, but asks for strong in vivo relevance of the findings, and clear experimental support of the major conclusions. Thus, we will not require addressing points regarding more mechanistic details experimentally. However, it will be necessary that in a revised manuscript you address all points questioning the main conclusions of the study, and all technical concerns, or points regarding the experimental designs, model systems used, or data presentation.

Given the constructive referee comments, I thus invite you to revise your manuscript with the understanding that all referee concerns must be addressed in the revised manuscript or in a detailed point-by-point response. Acceptance of your manuscript will depend on a positive outcome of a second round of review. It is EMBO reports policy to allow a single round of revision only and acceptance of the manuscript will therefore depend on the completeness of your responses included in the next, final version of the manuscript.

When submitting your revised manuscript, please also carefully review the instructions that follow below.

PLEASE NOTE THAT upon resubmission revised manuscripts are subjected to an initial quality control prior to exposition to re-review. Upon failure in the initial quality control, the manuscripts are sent back to the authors, which may lead to delays. Frequent reasons for such a failure are the lack of the data availability section (please see below) and the presence of statistics based on n=2 (the authors are then asked to present scatter plots or provide more data points).

When submitting your revised manuscript, we will require:

1) a .docx formatted version of the final manuscript text (including legends for main figures, EV figures and tables), but without the figures included. Please make sure that changes are highlighted to be clearly visible. Figure legends should be compiled at the end of the manuscript text.

2) individual production quality figure files as .eps, .tif, .jpg (one file per figure), of main figures and EV figures. Please upload these as separate, individual files upon re-submission. Please make sure that all figure panels are called out separately and sequentially in the manuscript text

The Expanded View format, which will be displayed in the main HTML of the paper in a collapsible format, has replaced the Supplementary information. You can submit up to 5 images as Expanded View. Please follow the nomenclature Figure EV1, Figure EV2 etc. The figure legend for these should be included in the main manuscript document file in a section called Expanded View Figure Legends after the main Figure Legends section. Additional Supplementary material should be supplied as a single pdf file labeled Appendix. The Appendix should have page numbers and needs to include a table of content on the first page (with page numbers) and legends for all content. Please follow the nomenclature Appendix Figure Sx, Appendix Table Sx etc. throughout the text, and also label the figures and tables according to this nomenclature.

For more details please refer to our guide to authors:
http://www.embopress.org/page/journal/14693178/authorguide#manuscriptpreparation

See also our guide for figure preparation:
http://wol-prod-cdn.literatumonline.com/pb-assets/embo-site/EMBOPress_Figure_Guidelines_061115-1561436025777.pdf

See also the guidelines for figure legend preparation:
https://www.embopress.org/page/journal/14693178/authorguide#figureformat

3) a .docx formatted letter INCLUDING the reviewers' reports and your detailed point-by-point responses to their comments. As part of the EMBO Press transparent editorial process, the point-by-point response is part of the Review Process File (RPF), which will be published alongside your paper.

4) a complete author checklist, which you can download from our author guidelines (https://www.embopress.org/page/journal/14693178/authorguide). Please insert page numbers in the checklist to indicate where the requested information can be found in the manuscript. The completed author checklist will also be part of the RPF.

Please also follow our guidelines for the use of living organisms, and the respective reporting guidelines:
http://www.embopress.org/page/journal/14693178/authorguide#livingorganisms

5) that primary datasets produced in this study (e.g. RNA-seq, ChIP-seq and array data) are deposited in an appropriate public database. This is now mandatory (like the COI statement). If no primary datasets have been deposited in any database, please state this in this section (e.g. 'No primary datasets have been generated and deposited').

See also: http://embor.embopress.org/authorguide#datadeposition

Please remember to provide a reviewer password if the datasets are not yet public.

The accession numbers and database should be listed in a formal "Data Availability " section (placed after Materials & Methods) that follows the model below. Please note that the Data Availability Section is restricted to new primary data that are part of this study.

Data availability

The datasets produced in this study are available in the following databases:

- RNA-Seq data: Gene Expression Omnibus GSE46843 (https://www.ncbi.nlm.nih.gov/geo/query/acc.cgi?acc=GSE46843)
- [data type]: [name of the resource] [accession number/identifier/doi] ([URL or identifiers.org/DATABASE:ACCESSION])

*** Note - All links should resolve to a page where the data can be accessed. ***

Moreover, I have these editorial requests:

6) We now request the publication of original source data with the aim of making primary data more accessible and transparent to the reader. Our source data coordinator will contact you to discuss which figure panels we would need source data for and will also provide you with helpful tips on how to upload and organize the files.

7) Our journal encourages inclusion of *data citations in the reference list* to directly cite datasets that were re-used and obtained from public databases. Data citations in the article text are distinct from normal bibliographical citations and should directly link to the database records from which the data can be accessed. In the main text, data citations are formatted as follows: "Data ref: Smith et al, 2001" or "Data ref: NCBI Sequence Read Archive PRJNA342805, 2017". In the Reference list, data citations must be labeled with "[DATASET]". A data reference must provide the database name, accession number/identifiers and a resolvable link to the landing page from which the data can be accessed at the end of the reference. Further instructions are available at: http://www.embopress.org/page/journal/14693178/authorguide#referencesformat

8) Regarding data quantification and statistics, please make sure that the number "n" for how many independent experiments were performed, their nature (biological versus technical replicates), the bars and error bars (e.g. SEM, SD) and the test used to calculate p-values is indicated in the respective figure legends (also for potential EV figures and all those in the final Appendix). Please also check that all the p-values are explained in the legend, and that these fit to those shown in the figure. Please provide statistical testing where applicable. Please avoid the phrase 'independent experiment', but clearly state if these were biological or technical replicates. Please also indicate (e.g. with n.s.) if testing was performed, but the differences are not significant. In case n=2, please show the data as separate datapoints without error bars and statistics.
See also:
http://www.embopress.org/page/journal/14693178/authorguide#statisticalanalysis

9) Please note our reference format:
http://www.embopress.org/page/journal/14693178/authorguide#referencesformat

10) We updated our journal's competing interests policy in January 2022 and request authors to consider both actual and perceived competing interests. Please review the policy https://www.embopress.org/competing-interests and add a statement declaring your competing interests. Please name that section 'Disclosure and Competing Interests Statement' and add it after the author contributions section.

11) Please order the manuscript sections like this using these names:
Title page - Abstract - Introduction - Results - Discussion - Materials and Methods - Data availability section (DAS) - Acknowledgements - Disclosure and Competing Interests Statement - References - Figure legends - Expanded View Figure legends

12) Please make sure that all the funding information is also entered into the online submission system and is complete and similar to the one in the manuscript text file (acknowledgements).

13) We now use CRediT to specify the contributions of each author in the journal submission system. CRediT replaces the author contribution section. Please use the free text box to provide more detailed descriptions. Thus, please remove the author

contributions section from the manuscript text file. See also guide to authors: https://www.embopress.org/page/journal/14693178/authorguide#authorshipguidelines

14) Please also add up to five keywords to the title page of the manuscript (below the abstract).

I look forward to seeing a revised version of your manuscript when it is ready. Please let me know if you have questions or comments regarding the revision.

Kind regards,

Achim

Achim Breiling
Senior Editor
EMBO Reports

Referee #1:

Papa and coworkers report a study of the effects of IP6 capsidation by HIV on innate sensing of virus in target cells. They show that the IP6 packaging-impaired mutant K158A exhibits a quantitatively stronger innate immune response than wild type HIV-1. The response is dampened by addition of the T8I mutation, which restores IP encapsidation into the K158A mutant virions. The authors also show evidence that TRIM5a is not involved in the sensing mechanism, but that STING is. Finally, the authors show that the effect can be observed in primary MDMs and in two human T cell lines (SupT1 and Jurkat).

While the experiments appear to have been performed well and the data are reasonable, my enthusiasm for this study is modest. The authors primary claim that the effect results from differential IP6 packaging is not sufficiently substantiated. While the T8I mutation rescues IP6 packaging in K158A particles, this mutation is known to affect the stability of the six-helix bundle in the immature lattice. While it is plausible that the observed effects are a consequence of IP6, it could result from an effect of the mutation unrelated to IP6 encapsidation. The authors have previously established that IP6 regulates HIV-1 maturation, so isn't the conclusion really that proper capsid assembly is important for cloaking the nascent viral DNA from detection? This doesn't seem like a great conceptual advance. I think that more could be done to understand what is going on with this mutant.

Specific concerns:
1. The authors have previously reported that DNA synthesis by K158A mutant virions is markedly impaired in target cells, as might be expected from a mutant with an unstable capsid. How then does DNA sensing occur? At a minimum, the authors should quantify the accumulation of early and late reverse transcripts of the wild type and mutant viruses in the target cells under the infection conditions used in these experiments. If the K158A capsid is unstable, then how much DNA is being synthesized? Is it being degraded in normal cells vs. STING knockout cells? Does ablation of DNA sensing (via cGAS or STING knockout) render cells more permissive to infection by K158A?

2. The use of RT as a normalizer of infection could be misleading since the K158A mutation markedly affects particle assembly. A thorough comparison of the protein composition of wild type and mutant particles would be useful here.

3. The conclusion regarding TRIM5a was drawn from a roundabout approach. I suggest the authors perform the experiment in TRIM5a knockout cells to answer the question directly.

4. In their 2021 Sci Adv. Paper, the authors reported that K158A infectivity is also rescued by N193H. Does the latter mutation restore IP6 incorporation and suppress innate immune detection?

5. Assays of capsid stability could also be helpful in interpreting the immune activation results.

Referee #2:

Papa and colleagues investigated the role of the IP6 polyaninon, which regulates HIV-1 capsid stability after entry in target cells, in the innate sensing of incoming virions. This is a relevant question as recent findings have revisited the role of the capsid in sensing, early trafficking, reverse-transcription and integration. These steps are tightly connected, allowing viral infection and escape of immune sensors. The authors hypothesize that an intact capsid may hide the viral genome from cell-intrinsic immunity. Using various viral mutants and cell culture systems, they propose that IP6 allows an escape of innate sensing by stabilizing the capsid in an intact form, triggering viral replication in T cell lines, THP-1 cells and primary macrophages. These results are of interest and in line with previous reports. Some claims deserve to be strengthened by additional controls.

Main comments

1. According to figures 1A and B, the K158A mutant mostly differs from other mutants by its proportion of "Mature (Multiple core)". Its profile is otherwise very close to the T8I mutant. A question to adress is whether "Mature (Multiple core)" may contain multiple viral genomes, and if this may be an alternative hypothesis to explain their increased sensing.
2. It is important to confirm that the decreased infectivity observed with some mutants is actually due to increased sensitivity to IFN by showing for instance a rescue of infection by using IFNAR-KO cells or treating the cells with anti-IFNAR antibodies.
3. Statistics should be added to figure 1B and the number of viruses analyzed indicated.
4. The requirement for cGAS and not TRIM5a for sensing could be confirmed with KO cells. The authors performed some experiments with SAMHD-1-KO THP1 cells. Could they use cGAS or Trim5a KO cells?
5. cGAS may localize in the cytoplasm or the nucleus. It would be interesting to discuss or provide data on the site of sensing of the K158A mutant.
6. Minor: Figure 1F is commented before figure 1E in the main text (lines 109 and 113)

Referee #3:

The manuscript by Papa et al. entitled "IP6-stabilised HIV capsids evade cGAS/STING-mediated host immune sensing" reports the ability of HIV-1 mutants, which are deficient for inositol hexakisphosphate (IP6) packaging, to activate immune signaling. IP6 is known to be abundant in cells and particularly enriched in HIV-1 particles, and to play an essential role in HIV-1 capsid formation and stability. IP6 is recruited to budding particles via two lysines (K158 and K227) and mutation of either of these lysines results in loss of IP6 packaging, defect in capsid formation and infectivity loss. In the present study, the authors showed that IP6-deficient viruses can be sensed by the cGAS-STING pathway in model cell lines and in primary monocyte-derived macrophages, a process that is dependent on de novo viral DNA synthesis. The manuscript is very well written and the results will certainly be of interest to the HIV-1 community. However, one must say that it wasn't completely unexpected that altering HIV-1 capsid stability would render the newly synthetized DNA more accessible to cellular sensors. Moreover, the manuscript would gain in strength if the following points were addressed.

Fig.1
The authors used HIV-1 WT viruses, the K158A mutant, which is known to alter capsid stability and collapses before completion of reverse transcription, the double K158A/T8I double mutant (as T8I mutation in SP1 partially restores that defect), and the T8I single mutant. The authors confirmed the expected phenotypes by cryoEM and infectivity assays.
However, in previous studies, the authors and others have reported some replication defect for the mutant T8I (PMID: 33692109; PMID 26537676), which was not observed here. Could the authors comment on this?

The authors used PMA-differentiated, SAMHD1-knockout THP-1 cells to assess the ability of these different viruses to induce innate immune sensing and showed a significant increase for K158A mutant as compared to WT, with an intermediary phenotype for K158A/T8I mutant, as measured by RT-qPCR on CXCL10, IFIT1, IFIT2 mRNAs and a larger panel of mRNAs, and on CXCL10 and TNFa cytokine production. Experiments with conditioned media confirmed that the cells infected with K158A mutant secreted antiviral cytokines.

Here, it would be essential here to measure interferon induction and production to complement the panel of analyzed cytokines. Considering that cells that were not KO for SAMHD1 were used in the following figures (e.g. Fig.3), one might wonder why these cells were used here in Fig.1 and what would be the results with WT THP-1 cells.

Fig.2
The authors then excluded a role of capsid-sensor TRIM5a by measuring the mRNAs for cytokines known to be induced by this sensor (and IL6 production in the supernatant) and observed no differences. A more direct way of checking this would be to generate TRIM5a KO cells.
Fig 2C. Of note, the mock condition is lacking to appreciate the basal production of IL-6 and should be added.

Using VLPs devoid of genome, the authors showed next that these cytokines were not specifically induced by the mutant, suggesting that sensing was TRIM5a-independent. Here the results would be strengthened by the use of TRIM5a KO cells (as mentioned above), as well as by measuring the panel of cytokines that the authors show as actually induced upon infection with genome-containing, mutant viruses in order to show that indeed VLPs don't induce them. A positive control for TRIM5a sensing is also lacking.

Fig 2B and F: the y-axis scale is really not appropriate and does not allow visualisation of the small effects; moreover, statistical analyses are lacking.

Fig.3
Next, the authors took advantage of PMA-treated, IFIT1-luc THP-1 cells that were KO for either STING or MAVS. The cells behaved as expected with respect to poly(I:C) and HT (herring testis)-DNA sensing (or lack thereof). IFIT1-luc induction was lost in STING KO but not in MAVS KO cells following infection with the K158A mutant. The use of H151 (a STING inhibitor), AZT (a

RT inhibitor) or a RT mutant respectively confirmed the data obtained with the KO cell lines and showed that de novo synthesized DNA was necessary to induce HIV-1 sensing.
Here a parallel control of the amounts of HIV-1 DNA accumulated in the different conditions over time would nicely complement the data. Indeed, as K158A is a 100-fold less infectious than WT (Fig. 1C and that was in SAMHD1 KO cells, not in the cells used here*), it would be interesting to measure the amounts of HIV-1 DNA synthesized over time in the different conditions and correlate this (or probably not) to the efficiency of sensing. Of note, (*) infectivity of the different viruses in the cell lines used here is lacking.

Fig.4
The authors then confirmed the data obtained in primary, monocyte-derived macrophages and T cell lines (Jurkat and SupT1 cells).
As for Fig.3, it would be essential to show the infectivity of the different viruses in these cells (as shown in Fig. 1C). Moreover, it would be highly interesting to test the phenotype in primary T cells even though they require activation to be permissive to HIV-1 infection.
As previously, interferons are lacking from the panel of cytokines and should be analyzed.

Finally, in addition to the capsid mutants, the authors could also use a pharmacological approach using PF96, which partially restores IP6 packaging, CA-SP1 cleavage and infectivity, in order to strengthen their conclusions.

Referee #1:

Q1. The authors have previously reported that DNA synthesis by K158A mutant virions is markedly impaired in target cells, as might be expected from a mutant with an unstable capsid. How then does DNA sensing occur? At a minimum, the authors should quantify the accumulation of early and late reverse transcripts of the wild type and mutant viruses in the target cells under the infection conditions used in these experiments. If the K158A capsid is unstable, then how much DNA is being synthesized? Is it being degraded in normal cells vs. STING knockout cells? Does ablation of DNA sensing (via cGAS or STING knockout) render cells more permissive to infection by K158A?

A1. We thank the reviewer for the suggestion to compare WT and K158A reverse transcription. To do this, we infected THP1 SAMHD1-KO cells and quantified RU5, GFP and 2ST which corresponds to early, middle and late transcripts respectively. Transcripts corresponding to every stage were detected for K158A but at substantially lower levels than WT. This correlates with the reduced infectivity of K158A. The fact that K158A stimulates a stronger immune response despite having lower detectable DNA levels is an excellent illustration of the importance of capsid protection. This new data therefore provides compelling new evidence supporting the manuscript findings and has been included as new figure panel 3H.

Q2. The use of RT as a normalizer of infection could be misleading since the K158A mutation markedly affects particle assembly. A thorough comparison of the protein composition of wild type and mutant particles would be useful here.

A1. To thoroughly quantify viral production, we compared RT activity measurements with p24 capsid protein levels and the absolute number of viral particles, as determined by TIRF microscopy. In each case, we observed that production of the mutant K158A was reduced compared to WT. There was particularly close correspondence between p24 and RT measurements, both of which are routinely used as methods for normalizing infection. This new data has been added as figures EV1B-E.

Q3. The conclusion regarding TRIM5a was drawn from a roundabout approach. I suggest the authors perform the experiment in TRIM5a knockout cells to answer the question directly.

A3. As the increased sensing of K158A was abolished in STING KO cells we did not feel it necessary to continue investigating TRIM5a. Nonetheless, two separate experiments suggest that K158A does not provoke increased TRIM5a sensing. First, there was no difference in IL-8 following infection with WT versus K158A, a cytokine whose induction has been shown to be dependent upon TRIM5 but not cGAS-STING. Second, when using VLPs that contain capsid but no genome, we did not observe increased sensing of K158A compared to WT. However, we appreciate that this VLP experiment rules out capsid sensing generally rather than TRIM5 specifically, so we have amended our manuscript to refer to ‘capsid sensing’ rather than ‘TRIM5 sensing’.

Q4. In their 2021 Sci Adv. Paper, the authors reported that K158A infectivity is also rescued by N193H. Does the latter mutation restore IP6 incorporation and suppress innate immune detection?

A4. There are a number of mutations that reduce IP6 incorporation and corresponding second-site mutants that restore incorporation and infectivity. As previous work has characterised these mutants in considerable detail and shown them to be functionally analogous, we would expect other combinations to behave like K158A and T8I. However, to allow us to go into as much depth as possible in testing the hypothesis that IP6 incorporation and capsid stability influences immune sensing we choose to focus on K158A and T8I as an exemplar mutant pair.

Q5. Assays of capsid stability could also be helpful in interpreting the immune activation results.

A5. We are grateful to the reviewer for this excellent suggestion. To link capsid stability with immune activation we used TIRF microscopy to compare WT and mutant virions used in our study. This new data, shown in Fig. 1C-D, nicely shows that K158A virions are unstable whilst addition of T8I rescues stability. With this new data we are able to further correlate decreased stability with increased sensing.

Referee #2:

Q1. According to figures 1A and B, the K158A mutant mostly differs from other mutants by its proportion of "Mature (Multiple core)". Its profile is otherwise very close to the T8I mutant. A question to adress is whether "Mature (Multiple core)" may contain multiple viral genomes, and if this may be an alternative hypothesis to explain their increased sensing.

A1. This is an interesting possibility and we have included this in our discussion of the new version of the manuscript (Lines 333-335), thank you.

Q2. It is important to confirm that the decreased infectivity observed with some mutants is actually due to

increased sensitivity to IFN by showing for instance a rescue of infection by using IFNAR-KO cells or treating the cells with anti-IFNAR antibodies. Could be other cytokines driving the block. (eg TNF)

A2. We agree with the reviewer that it may not be IFN driving the block to infection we see in our media conditioning experiment (Fig. 1M). Indeed, TNF production is substantially more increased compared to IFN production. Therefore, we reason that IFNAR-KO alone would not be expected to reverse the block to infection. It is also important to note that our study focused primarily at the triggering of sensing rather than the subsequent effector response. Follow-up studies will compare the contribution of induced effector responses to the reduced spreading infection of IP6-deficient viruses in different T cell lines.

Q3. Statistics should be added to figure 1B and the number of viruses analyzed indicated.

A3. We have indicated the number of viruses analysed for each mutant and calculated the sampling error for each category assuming a confidence level of 95%, as described previously (Mallery et al, 2021). The raw data plus sampling error has been provided as new Supplementary Figure EV1A.

Q4. The requirement for cGAS and not TRIM5a for sensing could be confirmed with KO cells. The authors performed some experiments with SAMHD-1-KO THP1 cells. Could they use cGAS or Trim5a KO cells?

A4. We decided to use STING KO cells rather than cGAS KO as these are functionally equivalent and STING is the obligate adaptor for cGAS. The ablation of immune detection by either the addition of AZT or the use of reverse transcriptase mutant D185E confirms that K158A detection is dependent upon sensing of viral DNA, while the use of capsid-only VLPs shows that capsid detection, such as by TRIM5a, is not involved.

Q5. cGAS may localize in the cytoplasm or the nucleus. It would be interesting to discuss or provide data on the site of sensing of the K158A mutant.

A5. We agree that this is an interesting question and something we would like to address in future work. Several reports suggest that nuclear cGAS is inactive when it’s bound to chromatin in the nucleus. In contrast, it has been proposed that HIV2 cDNA is detected by cGAS in the nucleus (Lahaye et al 2018), although it was unclear in this study whether nuclear sensing of HIV1 dsDNA also occurred. Distinguishing between cytoplasmic and nuclear sensing as opposed to merely binding is likely to be technically challenging however. Nevertheless, to address this possibility in the current study we have added the following to the discussion (line 350): “*Our study is also in line with a previous report (Sumner et al, 2020) showing that when Gag processing is perturbed by protease inhibition this leads to increased sensing. Where exactly unstable capsids lose integrity and are sensed remains to be determined. Given that HIV-2 cGAS sensing has been reported in the nucleus (Lahaye et al. 2018), one possible approach to address this may be to determine whether sensing is driven by cGAS localised in the cytoplasm or nucleus*”.

Q6. Minor: Figure 1F is commented before figure 1E in the main text (lines 109 and 113)

A6. The order of the figures has been amended.

Referee #3:

Q1. Fig.1:The authors used HIV-1 WT viruses, the K158A mutant, which is known to alter capsid stability and collapses before completion of reverse transcription, the double K158A/T8I double mutant (as T8I mutation in SP1 partially restores that defect), and the T8I single mutant. The authors confirmed the expected phenotypes by cryoEM and infectivity assays. However, in previous studies, the authors and others have reported some replication defect for the mutant T8I (PMID: 33692109; PMID 26537676), which was not observed here. Could the authors comment on this?

A1. We thank the reviewer for pointing this out. In repeating the experiment using a virus titration we realised that the previous dataset had exceeded the linear range of the experiment, resulting in the modest T8I defect being undetected. We have therefore replaced the data in the previous Figure 1C with a titration (now Fig. 1E), in which a similar defect to that previously reported can be seen. Our apologies for this error.

Q2. The authors used PMA-differentiated, SAMHD1-knockout THP-1 cells to assess the ability of these different viruses to induce innate immune sensing and showed a significant increase for K158A mutant as compared to WT, with an intermediary phenotype for K158A/T8I mutant, as measured by RT-qPCR on CXCL10, IFIT1, IFIT2 mRNAs and a larger panel of mRNAs, and on CXCL10 and TNFa cytokine production. Experiments with conditioned media confirmed that the cells infected with K158A mutant secreted antiviral cytokines. Here, it would be essential here to measure interferon induction and production to complement the panel of analyzed cytokines.

A2. We thank the reviewer for this suggestion and have evaluated a panel of human interferons, the data for which has been included as a new figure panel, Fig. 1K.

Q3. Considering that cells that were not KO for SAMHD1 were used in the following figures (e.g. Fig.3), one might wonder why these cells were used here in Fig.1 and what would be the results with WT THP-1 cells.

A3. We thank the reviewer for this suggestion. We have added data for sensing in WT THP1s as Figure EV1F. K158A induced a significantly greater CXCL10 and IFIT1 response than WT, though the magnitude of response was smaller than in SAMHD1 KO cells. We therefore used SAMHD1 KOs for some experiments to benefit from the greater signal.

Q4. Fig.2. The authors then excluded a role of capsid-sensor TRIM5a by measuring the mRNAs for cytokines known to be induced by this sensor (and IL6 production in the supernatant) and observed no differences. A more direct way of checking this would be to generate TRIM5a KO cells.

A4. We decided to use capsid-only VLPs rather than TRIM5a KOs as with this approach we could rule out any kind of capsid sensing, not just TRIM5. K158A VLPs did not induce a significantly increased immune response compared to WT, indicating that a capsid sensor was not involved. In parallel, STING KO and inhibitor experiments plus the use of AZT and a reverse transcriptase mutant all show that sensing is dependent upon viral DNA. We agree that if our immune sensing phenotype for K158A had not been clearly DNA-dependent then a TRIM5a KO would have been a great next step. However, given that we did not perform a TRIM5 KO experiment, we have amended both our abstract and the relevant results section to more properly refer to 'capsid detection' rather than 'TRIM5 detection', in line with our use of VLPs.

Q5. Fig 2C. Of note, the mock condition is lacking to appreciate the basal production of IL-6 and should be added.

A5. IL-6 quantification in mock condition has been added in the new Fig. 2F as suggested.

Q6. Using VLPs devoid of genome, the authors showed next that these cytokines were not specifically induced by the mutant, suggesting that sensing was TRIM5a-independent. Here the results would be strengthened by the use of TRIM5a KO cells (as mentioned above), as well as by measuring the panel of cytokines that the authors show as actually induced upon infection with genome-containing, mutant viruses in order to show that indeed VLPs don't induce them. A positive control for TRIM5a sensing is also lacking.

A6. As discussed above, we have amended the description of our VLP experiments to more properly refer to 'capsid sensing' rather than 'TRIM5a sensing' (Line 180-182). As regards a positive control, the data in Figure 2D for the VLPs shows that there is detection of capsid and therefore some sensing, but it is not significantly different between WT and K158A. Further evidence that we are measuring capsid sensing (positive control) is included in Figure 2E where we detect responses during infection of genome-containing particles. While both WT and K158A are detected there is no difference in cytokines associated with TRIM5a.

Q7. Fig 2B and F: the y-axis scale is really not appropriate and does not allow visualisation of the small effects; moreover, statistical analyses are lacking.

A7. The y-axis scale has been changed and statistical analysis has been added as suggested and are present now in the new Fig 2D and E.

Q8. Fig.3. Next, the authors took advantage of PMA-treated, IFIT1-luc THP-1 cells that were KO for either STING or MAVS. The cells behaved as expected with respect to poly(I:C) and HT (herring testis)-DNA sensing (or lack thereof). IFIT1-luc induction was lost in STING KO but not in MAVS KO cells following infection with the K158A mutant. The use of H151 (a STING inhibitor), AZT (a RT inhibitor) or a RT mutant respectively confirmed the data obtained with the KO cell lines and showed that de novo synthesized DNA was necessary to induce HIV-1 sensing.
Here a parallel control of the amounts of HIV-1 DNA accumulated in the different conditions over time would nicely complement the data. Indeed, as K158A is a 100-fold less infectious than WT (Fig. 1C and that was in SAMHD1 KO cells, not in the cells used here), it would be interesting to measure the amounts of HIV-1 DNA synthesized over time in the different conditions and correlate this (or probably not) to the efficiency of sensing. Of note, (*) infectivity of the different viruses in the cell lines used here is lacking.*

A8. We thank the reviewer for the idea to complement our DNA sensing data with measurements of reverse transcription inside cells. We have performed the suggested experiment and quantified the amount of early, middle and late dsDNA viral transcripts in SAMHD1-KO cells upon infection with different HIV mutants. K158A mutant retrotranscribed its genome, although with a lower efficiency compared to WT and K158A/T8I. This result is reassuring as it shows that we are not driving increased sensing of K158A simply because there is more virus and thus more retrotranscribed DNA. More importantly, it suggests that the context of newly transcribed viral

DNA rather than absolute quantity is a critical determinant of immune sensing. This result also fits with the other new data included in our revised manuscript showing that K158A capsids are unstable.

Q9. Fig.4. The authors then confirmed the data obtained in primary, monocyte-derived macrophages and T cell lines (Jurkat and SupT1 cells). As for Fig.3, it would be essential to show the infectivity of the different viruses in these cells (as shown in Fig. 1C).

A9. Infectivity data for the different cell lines (SupT1, Jurkat) used has now been added as Fig EV4A-B as suggested.

Q10. Moreover, it would be highly interesting to test the phenotype in primary T cells even though they require activation to be permissive to HIV-1 infection.

A10. We agree with the reviewer that testing the phenotype in primary T cells would be interesting, however, this is beyond our scope for the current paper.

Q11. As previously, interferons are lacking from the panel of cytokines and should be analyzed.

A11. We have now analysed a range of interferons and added this data to Fig. 1K, 4A, 4E, 4I. Different interferon genes were evaluated in the different cell lines and added to the panel of cytokines as suggested (Fig.4). Several IFN stimulated genes were upregulated during infection with K158A reflecting a strong antiviral state stimulated by IP6-defective particles.

Q12. Finally, in addition to the capsid mutants, the authors could also use a pharmacological approach using PF96, which partially restores IP6 packaging, CA-SP1 cleavage and infectivity, in order to strengthen their conclusions.

A12. How maturation inhibitors (MIs) such as PF96 influence immune detection of HIV-1 is a really interesting question and one we are planning to explore systematically in a separate study. Given that MIs alter capsid processing in addition to influencing IP6 packaging, we anticipate that this will give rise to complex phenotypes. We plan to compare different MIs and investigate whether second-site escape mutants restore immune evasion and whether this influences reported changes in replication kinetics in T cell lines.

Dear Dr. James,

Thank you for the submission of your revised manuscript to our editorial offices. I have now received the reports from the three referees that I asked to re-evaluate your study, you will find below. As you will see, the referees now support the publication of your study in EMBO reports. However, referee #1 has some comments and suggestions to improve the manuscript, I ask you to address in a final revise manuscript. Please also provide a final p-b-p-response addressing these remaining points.

Moreover, I have these editorial requests I also ask you to address:

- We plan to publish your manuscript in the Report format (as also indicated by you in the submission system), as there are not more than 5 main figures. For a Scientific Report we require that results and discussion sections are combined in a single chapter called "Results & Discussion". Please do this for your manuscript. For more details, please refer to our guide to authors: http://www.embopress.org/page/journal/14693178/authorguide#researcharticleguide

- Please add up to five keywords to the manuscript text, below the abstract.

- Please add a data availability section to the manuscript text and move the information on deposited datasets (mass spectrometry proteomics) there. Please remove the referee token from the data availability section, add a direct link to the dataset and make sure that the dataset is public latest upon online publication of the study.

- We updated our journal's competing interests policy in January 2022 and request authors to consider both actual and perceived competing interests. Please review the policy https://www.embopress.org/competing-interests and update your competing interests if necessary. Please name this section 'Disclosure and Competing Interests Statement' and put it after the Acknowledgements section.

- Please add a formal 'Data Availability' section (DAS) to the manuscript, also if no large datasets have been submitted to a public database. Please state there 'No large primary datasets have been generated and deposited'.

- Please order the manuscript sections like this (using these names as headings):
Title page - Abstract - Key Words - Introduction - Results & Discussion - Materials and Methods - Data availability section - Acknowledgements - Disclosure and Competing Interests Statement - References - Figure legends - Expanded View Figure Legends

- Regarding data quantification and statistics, please make sure that the number "n" for how many independent experiments were performed, their nature (biological versus technical replicates), the bars and error bars (e.g. SEM, SD) and the test used to calculate p-values is indicated in the respective figure legends (also for potential EV figures and all those in the final Appendix). Please also check that all the p-values are explained in the legend, and that these fit to those shown in the figure. Please provide statistical testing where applicable. Please avoid the phrase 'independent experiment', but clearly state if these were biological or technical replicates. Please also indicate (e.g. with n.s.) if testing was performed, but the differences are not significant. In case n=2, please show the data as separate datapoints without error bars and statistics. See also: http://www.embopress.org/page/journal/14693178/authorguide#statisticalanalysis

If n<5, please show single datapoints for diagrams. Presently, it seems there are diagrams without or only with partial statistics.

- Please name Figure EV4 'Figure EV2' and correct all the callouts.

- Thanks for providing the source data. Please upload this as one pdf file per figure or as one folder with all the source data files for one figure ZIPed together.

- Finally, please find attached a word file of the manuscript text (provided by our publisher) with changes we ask you to include in your final manuscript text and comments. Please use the attached file as basis for further revisions and provide your final manuscript file with track changes, in order that we can see any modifications done.

In addition, I would need from you:
- a short, two-sentence summary of the manuscript (not more than 35 words).
- two to four short bullet points highlighting the key findings of your study (two lines each).
- a schematic summary figure that provides a sketch of the major findings (not a data image) in jpeg or tiff format (with the exact width of 550 pixels and a height of not more than 400 pixels) that can be used as a visual synopsis on our website.

I look forward to seeing the final revised version of your manuscript when it is ready. Please let me know if you have questions regarding the revision.

Please use this link to submit your revision: https://embor.msubmit.net/cgi-bin/main.plex

Best,

Achim Breiling
Senior Editor
EMBO Reports

Referee #1:

The authors have submitted a revised manuscript with most of my criticisms thoroughly addressed. The study shows: (1) IP6 encapsidation is important for formation of a sufficiently stable capsid, and (2) a stable capsid helps protect the nascent DNA from sensing. Both conclusions have been drawn in previous studies.

I offer the following suggestions for improvement.

1. Conceptual: Actually, it is not known that IP6 in the virion stabilizes the capsid. It is possible that IP6 promotes formation an architecturally correct capsid of proper stability, but once the capsid is formed, IP6's job is done. This should be discussed.
2. Fig. 1, C&D: Would the addition of IP6 stabilize the K158A mutant capsid? The answer to this question may establish whether the mutant capsid is defective because it cannot bind IP6 or because it did not incorporate it. This may bolster the authors' argument that this mutant undergoes DNA sensing because it doesn't have IP6 in the particles.
3. It is possible that target cell IP6 is responsible for the observed reverse transcription by the mutant in target cells, but the particles fall apart owing to architectural defects. Can the permeabilized virions undergo reverse transcription in vitro in reactions containing IP6? Does depletion of IP6 in target cells squelch K158A-dependent activation?
4. Fig. 3H: the viruses used in this type of experiment often contain significant quantities of contaminating plasmid DNA. Therefore, the authors must include appropriate controls (e.g., parallel infections containing an RT inhibitor) to determine the level of background for each virus.
5. Line 326 states: "Second, any capsids that do form will lack IP6 to stabilize them and rapidly collapse when no longer enclosed in the viral membrane." While consistent with in vitro data, this statement ignores the possibility that IP6 in target cells could stabilize incoming capsids lacking IP6.
6. Line 363 states: "having charged pores for dNTP import creates the requirement for IP6: positively charged pores are needed to import negatively charged dNTPs, resulting in charge repulsion that needs to be neutralized by a negatively charged molecule like IP6." I am skeptical about the requirement for a charged pore to import dNTPs. The study in which this conclusion was drawn (Jacques et al., 2016) was flawed because the mutants that were studied had unstable capsids, possibly owing to impaired IP6 binding.

Minor/Textual:

7. Abstract final sentence: "These results demonstrate that IP6 is not only required to build capsids that can successfully transit the cell but also avoid host innate immune sensing." This sentence can be improved. I suggest the following: These results demonstrate that IP6 is required to build capsids that can successfully transit the cell and avoid host innate immune sensing.
8. On line 320, the authors state: "By stabilizing the 6HB, T8I restores the ability of K158A to package IP6, thereby rescuing mature capsid formation and infection (Mallery et al, 2021). The "thereby" implies a cause-effect relationship between IP6 packaging and mature capsid formation. While this is plausible, the mechanism by which IP6 promotes mature capsid formation, and whether it is required following maturation, are unknown. A more thoughtful discussion regarding these unanswered questions would be appreciated.
9. "Data" is a plural noun. Please write "these data suggest..."
10. Lines 668-673: duplicated reference.

Referee #2:

The authors have addressed all my previous concerns.

Referee #3:

The authors have addressed my concerns.

Referee #1:

The authors have submitted a revised manuscript with most of my criticisms thoroughly addressed. The study shows: (1) IP6 encapsidation is important for formation of a sufficiently stable capsid, and (2) a stable capsid helps protect the nascent DNA from sensing. Both conclusions have been drawn in previous studies.

I offer the following suggestions for improvement.

1. Conceptual: Actually, it is not known that IP6 in the virion stabilizes the capsid. It is possible that IP6 promotes formation an architecturally correct capsid of proper stability, but once the capsid is formed, IP6's job is done. This should be discussed.

We thank the reviewer for this suggestion. We have expanded the discussion to summarise the published data on IP6 and its roles in assembly and stability. For the latter we refer to in vitro TIRF and ERT assays that demonstrate that the capsid is intrinsically unstable in the absence of IP6, suggesting that IP6 is still needed once the capsid has formed. However, we also make clear that reducing IP6 levels in cells through kinase knockout has not been shown to reduce the infectivity of wild-type HIV-1.

2. Fig. 1, C&D: Would the addition of IP6 stabilize the K158A mutant capsid? The answer to this question may establish whether the mutant capsid is defective because it cannot bind IP6 or because it did not incorporate it. This may bolster the authors' argument that this mutant undergoes DNA sensing because it doesn't have IP6 in the particles.

The TIRF microscopy-based virion stability experiments (Fig 1C-D) were carried out in the presence of IP6 and show that K158A collapses even when IP6 is included in the reaction, unlike wild-type virus. This implies that the K158A capsid is intrinsically less stable than the wild-type, consistent with this capsid mutant being more susceptible to immune sensing due to its inability to protect double-stranded DNA during reverse transcription.
We have added a sentence to clarify that the TIRF assay was performed in the presence of IP6 (line 111). We have also indicated the concentration of IP6 used in these experiments in the M&M section (line 526).
As the data indicates K158A has both an assembly and stability defect, we have indicated that either could be the cause of DNA sensing: "Taken together, this suggests that sensing is caused by incomplete K158A capsids entering cells and reverse transcribing or complete capsids that undergo partial DNA synthesis and then collapse before reaching the nucleus.".

3. It is possible that target cell IP6 is responsible for the observed reverse transcription by the mutant in target cells, but the particles fall apart owing to architectural defects. Can the permeabilized virions undergo reverse transcription in vitro in reactions containing IP6?

Combining ERT and TIRF in a single assay would be a powerful approach but unfortunately not something we are technically able to do at present. However, our cellular data shows that K158A capsids are capable of undergoing reverse transcription under physiological IP6 levels, albeit less viral DNA is detected than for wild-type.

Does depletion of IP6 in target cells squelch K158A-dependent activation?

We thank the reviewer for the question. We have added infectivity data of K158A in cells depleted of IP6 (IPMK-KO and IPPK-KO) (Fig EV2D), showing that there is no reduction in K158A infectivity in such cells, suggests that the concentration of IP6 is still well above that of the capsid K_D. Thus, we would not expect to see any impact on immune sensing. Text was modified as follow (line 121): "Additionally, the low infectivity of the K158 mutant was not affected by the removal of IP6 from target cells lacking IPPK or IPMK enzymes (EV1B) (Mallery et al. 2019), suggesting that the concentration of IP6 is still well above that of the capsid K_D"

4. Fig. 3H: the viruses used in this type of experiment often contain significant quantities of contaminating plasmid DNA. Therefore, the authors must include appropriate controls (e.g., parallel infections containing an RT inhibitor) to determine the level of background for each virus.

We have added data to show the background level for each virus, using cells that are infected with boiled inactivated virus. This has been added as a panel in figure EV2. We have also modified the text accordingly (line 268): 'To determine the level of background contamination, for instance from plasmid DNA, the levels of the RU5, GFP and ST transcripts were also quantified in cells infected with boiled-inactivated virus (Fig.EV2A)'

5. Line 326 states: "Second, any capsids that do form will lack IP6 to stabilize them and rapidly collapse when no longer enclosed in the viral membrane." While consistent with in vitro data, this statement ignores the possibility that IP6 in target cells could stabilize incoming capsids lacking IP6.

We have amended this section as follows to address this possibility (Line 374): "Second, any capsids that do form will lack IP6 to stabilize them and are likely to rapidly collapse when no longer enclosed in the viral membrane. Even if there is IP6 available in target cells, there are hundreds of binding sites per capsid and collapse may occur before reaching IP6:capsid equilibrium.".

6. Line 363 states: "having charged pores for dNTP import creates the requirement for IP6: positively charged pores are needed to import negatively charged dNTPs, resulting in charge repulsion that needs to be neutralized by a negatively charged molecule like IP6." I am skeptical about the requirement for a charged pore to import dNTPs. The study in which this conclusion was drawn (Jacques et al., 2016) was flawed because the mutants that were studied had unstable capsids, possibly owing to impaired IP6 binding.

We have amended this sentence to clarify that this is our own speculation (Line 425): "However, as this is not the only way to build and stabilise a viral capsid, we speculate IP6 is needed because of the charged pores, rather than vice versa...".

Minor/Textual:

7. Abstract final sentence: "These results demonstrate that IP6 is not only required to build capsids that can successfully transit the cell but also avoid host innate immune sensing." This sentence can be improved. I suggest the following: These results demonstrate that IP6 is required to build capsids that can successfully transit the cell and avoid host innate immune sensing.

We thank the reviewer for the suggestion and the text has been modified accordingly.

8. On line 320, the authors state: "By stabilizing the 6HB, T8I restores the ability of K158A to package IP6, thereby rescuing mature capsid formation and infection (Mallery et al, 2021). The "thereby" implies a cause-effect relationship between IP6 packaging and mature capsid formation. While this is plausible, the mechanism by which IP6 promotes mature capsid formation, and whether it is required following maturation, are unknown. A more thoughtful discussion regarding these unanswered questions would be appreciated.

We agree with the reviewer and the sentence has been amended in the text. We have also expanded the discussion around IP6 packaging in virions and mature capsid assembly.

9. "Data" is a plural noun. Please write "these data suggest..."

This has been amended in the text.

10. Lines 668-673: duplicated reference.

We thank the reviewer for spotting this. The duplicated reference has been removed.

In addition, I would need from you:
- a short, two-sentence summary of the manuscript (not more than 35 words).

HIV-1 uses IP6 to build a capsid inside which it can synthesise DNA while avoiding detection by cGAS/STING-mediated DNA sensing. Capsids lacking IP6 become unstable, expose viral DNA and alert host immunity.

- two to four short bullet points highlighting the key findings of your study (two lines each).

- IP6-stabilised HIV capsids hide viral DNA from innate immune detection.

- HIV-1 virions that incorporate insufficient IP6 during production trigger a host immune response when they infect macrophages and T cells.

- IP6-deficient HIV particles have unstable capsids that expose newly synthesised viral DNA to the cellular DNA sensor/adaptor cGAS/STING.

- a schematic summary figure that provides a sketch of the major findings (not a data image) in jpeg or tiff format (with the exact width of 550 pixels and a height of not more than 400 pixels) that can be used as a visual synopsis on our website.

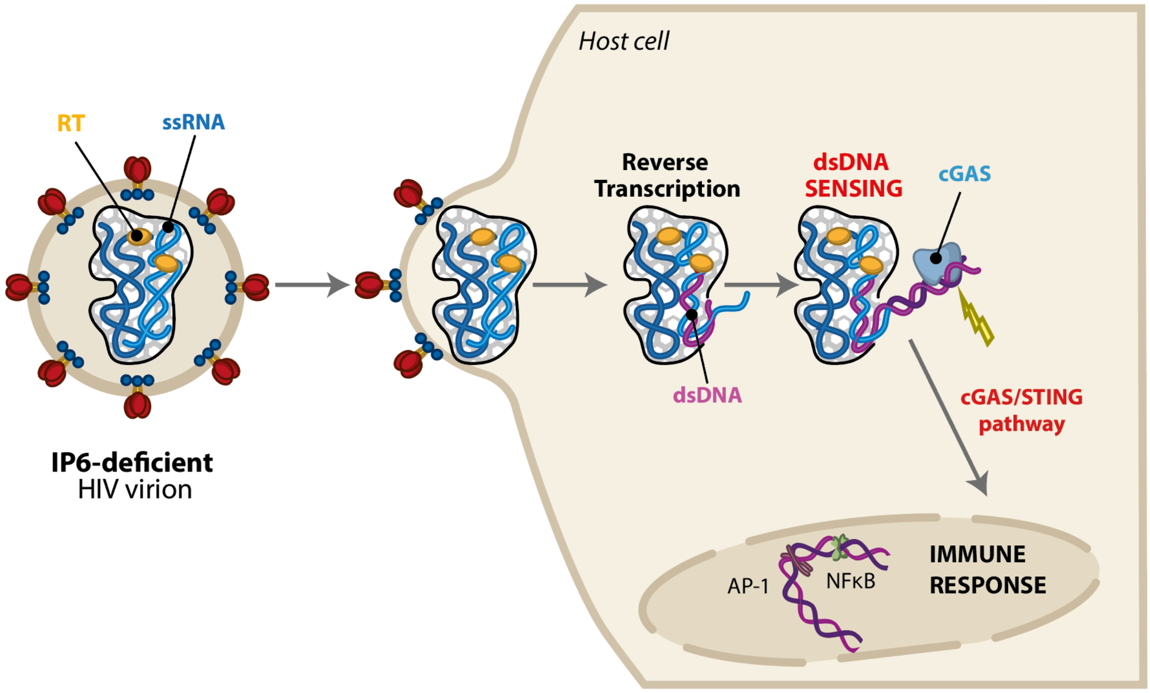

Dear Dr. James,

Thank you for the submission of your revised manuscript to our editorial offices. I now went through this and your p-b-p-response, and I consider the remaining referee concerns as adequately addressed.

However, I have these further editorial requests:

- We now request to show single datapoints for all diagrams with n<5. Please do that for all the diagrams shown. I.e. if n=3, the three data points should be shown together with the mean/medium and the error bars.

- For several diagrams you indicate n=2. In these cases, please show the data as separate datapoints or bars without error bars and statistics. For all diagrams with n=3 and more, please make sure that full statistics is shown.

- Please add information on the size of the scale bars in Fig. 1A to the legend.

- Please add a paragraph to the methods section (titled 'Biosafety') providing details on where and how biosafety-relevant experiments with viruses were performed and that these were approved, and by whom (institution, government).

I look forward to seeing the final revised version of your manuscript when it is ready. Please let me know if you have questions regarding the revision.

Please use this link to submit your revision: https://embor.msubmit.net/cgi-bin/main.plex

Best,

Achim Breiling
Senior Editor
EMBO Reports

The authors have addressed all minor editorial requests.

3rd Revision - Editorial Decision 10th Mar 2023

Dr. Leo James
MRC Laboratory of Molecular Biology
PNAC
Hills Road
Cambridge, Cambridgeshire CB2 2QH
United Kingdom

Dear Dr. James,

I am very pleased to accept your manuscript for publication in the next available issue of EMBO reports. Thank you for your contribution to our journal.

At the end of this email I include important information about how to proceed. Please ensure that you take the time to read the information and complete and return the necessary forms to allow us to publish your manuscript as quickly as possible.

As part of the EMBO publication's Transparent Editorial Process, EMBO reports publishes online a Review Process File to accompany accepted manuscripts. As you are aware, this File will be published in conjunction with your paper and will include the referee reports, your point-by-point response and all pertinent correspondence relating to the manuscript.

If you do NOT want this File to be published, please inform the editorial office within 2 days, if you have not done so already, otherwise the File will be published by default [contact: emboreports@embo.org]. If you do opt out, the Review Process File link will point to the following statement: "No Review Process File is available with this article, as the authors have chosen not to make the review process public in this case."

Thank you again for your contribution to EMBO reports and congratulations on a successful publication. Please consider us again in the future for your most exciting work.

Yours sincerely,

Achim Breiling
Editor
EMBO Reports

****

THINGS TO DO NOW:

Please note that you will be contacted by Wiley Author Services to complete licensing and payment information. The required 'Page Charges Authorization Form' is available here: https://www.embopress.org/pb-assets/embo-site/er_apc.pdf - please download and complete the form and return to embopressproduction@wiley.com

EMBO Press participates in many Publish and Read agreements that allow authors to publish Open Access with reduced/no publication charges. Check your eligibility: https://authorservices.wiley.com/author-resources/Journal-Authors/open-access/affiliation-policies-payments/index.html

You will receive proofs by e-mail approximately 2-3 weeks after all relevant files have been sent to our Production Office; you should return your corrections within 2 days of receiving the proofs.

Please inform us if there is likely to be any difficulty in reaching you at the above address at that time. Failure to meet our deadlines may result in a delay of publication, or publication without your corrections.

All further communications concerning your paper should quote reference number EMBOR-2022-56275V4 and be addressed to emboreports@wiley.com.

Should you be planning a Press Release on your article, please get in contact with emboreports@wiley.com as early as possible, in order to coordinate publication and release dates.

EMBO Press Author Checklist

Corresponding Author Name: Leo James	
Journal Submitted to: EMBO REPORTS	
Manuscript Number: EMBOR-2022-56275V2	

USEFUL LINKS FOR COMPLETING THIS FORM
The EMBO Journal - Author Guidelines
EMBO Reports - Author Guidelines
Molecular Systems Biology - Author Guidelines
EMBO Molecular Medicine - Author Guidelines

Reporting Checklist for Life Science Articles (updated January 2022)
This checklist is adapted from Materials Design Analysis Reporting (MDAR) Checklist for Authors. MDAR establishes a minimum set of requirements in transparent reporting in the life sciences (see Statement of Task: 10.31222/osf.io/9sm4x). Please follow the journal's guidelines in preparing your manuscript.
Please note that a copy of this checklist will be published alongside your article.

Abridged guidelines for figures
1. Data
The data shown in figures should satisfy the following conditions:
- the data were obtained and processed according to the field's best practice and are presented to reflect the results of the experiments in an accurate and unbiased manner.
- ideally, figure panels should include only measurements that are directly comparable to each other and obtained with the same assay.
- plots include clearly labeled error bars for independent experiments and sample sizes. Unless justified, error bars should not be shown for technical replicates.
- if n<5, the individual data points from each experiment should be plotted. Any statistical test employed should be justified.
- Source Data should be included to report the data underlying figures according to the guidelines set out in the authorship guidelines on Data Presentation.

2. Captions
Each figure caption should contain the following information, for each panel where they are relevant:
- a specification of the experimental system investigated (eg cell line, species name).
- the assay(s) and method(s) used to carry out the reported observations and measurements.
- an explicit mention of the biological and chemical entity(ies) that are being measured.
- an explicit mention of the biological and chemical entity(ies) that are altered/varied/perturbed in a controlled manner.
- the exact sample size (n) for each experimental group/condition, given as a number, not a range;
- a description of the sample collection allowing the reader to understand whether the samples represent technical or biological replicates (including how many animals, litters, cultures, etc.).
- a statement of how many times the experiment shown was independently replicated in the laboratory.
- definitions of statistical methods and measures:
  - common tests, such as t-test (please specify whether paired vs. unpaired), simple χ2 tests, Wilcoxon and Mann-Whitney tests, can be unambiguously identified by name only, but more complex techniques should be described in the methods section;
  - are tests one-sided or two-sided?
  - are there adjustments for multiple comparisons?
  - exact statistical test results, e.g., P values = x but not P values < x;
  - definition of 'center values' as median or average;
  - definition of error bars as s.d. or s.e.m.

Please complete ALL of the questions below.
Select "Not Applicable" only when the requested information is not relevant for your study.

Materials

Newly Created Materials	Information included in the manuscript?	In which section is the information available? (Reagents and Tools Table, Materials and Methods, Figures, Data Availability Section)
New materials and reagents need to be available; do any restrictions apply?	Yes	Materials and Method

Antibodies	Information included in the manuscript?	In which section is the information available? (Reagents and Tools Table, Materials and Methods, Figures, Data Availability Section)
For **antibodies** provide the following information:<br>- Commercial antibodies: RRID (if possible) or supplier name, catalogue number and or/clone number<br>- Non-commercial: RRID or citation	Not Applicable	

DNA and RNA sequences	Information included in the manuscript?	In which section is the information available? (Reagents and Tools Table, Materials and Methods, Figures, Data Availability Section)
**Short novel DNA or RNA including primers, probes**: provide the sequences.	Yes	Materials and Method

Cell materials	Information included in the manuscript?	In which section is the information available? (Reagents and Tools Table, Materials and Methods, Figures, Data Availability Section)
**Cell lines:** Provide species information, strain. Provide accession number in repository **OR** supplier name, catalog number, clone number, and/**OR** RRID.	Yes	Materials and Method
**Primary cultures:** Provide species, strain, sex of origin, genetic modification status.	Not Applicable	
Report if the cell lines were recently **authenticated** (e.g., by STR profiling) and tested for mycoplasma contamination.	Not Applicable	

Experimental animals	Information included in the manuscript?	In which section is the information available? (Reagents and Tools Table, Materials and Methods, Figures, Data Availability Section)
**Laboratory animals or Model organisms:** Provide species, strain, sex, age, genetic modification status. Provide accession number in repository **OR** supplier name, catalog number, clone number, **OR** RRID.	Not Applicable	
**Animal observed in or captured from the field:** Provide species, sex, and age where possible.	Not Applicable	
Please detail **housing and husbandry conditions**.	Not Applicable	

Plants and microbes	Information included in the manuscript?	In which section is the information available? (Reagents and Tools Table, Materials and Methods, Figures, Data Availability Section)
**Plants:** provide species and strain, ecotype and cultivar where relevant, unique accession number if available, and source (including location for collected wild specimens).	Not Applicable	
**Microbes:** provide species and strain, unique accession number if available, and source.	Not Applicable	

Human research participants	Information included in the manuscript?	In which section is the information available? (Reagents and Tools Table, Materials and Methods, Figures, Data Availability Section)
If collected and within the bounds of privacy constraints report on age, sex and gender or ethnicity for all study participants.	Not Applicable	

Core facilities	Information included in the manuscript?	In which section is the information available? (Reagents and Tools Table, Materials and Methods, Figures, Data Availability Section)
If your work benefited from core facilities, was their service mentioned in the acknowledgments section?	Not Applicable	

Design

Study protocol	Information included in the manuscript?	In which section is the information available? (Reagents and Tools Table, Materials and Methods, Figures, Data Availability Section)
If study protocol has been **pre-registered, provide DOI in the manuscript**. For clinical trials, provide the trial registration number **OR** cite DOI.	Not Applicable	
Report the **clinical trial registration number** (at ClinicalTrials.gov or equivalent), where applicable.	Not Applicable	

Laboratory protocol	Information included in the manuscript?	In which section is the information available? (Reagents and Tools Table, Materials and Methods, Figures, Data Availability Section)
Provide DOI OR other citation details if **external detailed step-by-step protocols** are available.	Not Applicable	

Experimental study design and statistics	Information included in the manuscript?	In which section is the information available? (Reagents and Tools Table, Materials and Methods, Figures, Data Availability Section)
Include a statement about **sample size** estimate even if no statistical methods were used.	Not Applicable	
Were any steps taken to minimize the effects of subjective bias when allocating animals/samples to treatment (e.g. **randomization procedure**)? If yes, have they been described?	Not Applicable	
Include a statement about **blinding** even if no blinding was done.	Not Applicable	
Describe **inclusion/exclusion criteria** if samples or animals were excluded from the analysis. Were the criteria pre-established?<br><br>If sample or data points were omitted from analysis, report if this was due to attrition or intentional exclusion and provide justification.	Not Applicable	
For every figure, are **statistical tests** justified as appropriate? Do the data meet the assumptions of the tests (e.g., normal distribution)? Describe any methods used to assess it. Is there an estimate of variation within each group of data? Is the variance similar between the groups that are being statistically compared?	Not Applicable	

Sample definition and in-laboratory replication	Information included in the manuscript?	In which section is the information available? (Reagents and Tools Table, Materials and Methods, Figures, Data Availability Section)
In the figure legends: state number of times the experiment was **replicated** in laboratory.	Yes	
In the figure legends: define whether data describe **technical or biological replicates**.	Yes	

Ethics

Ethics	Information included in the manuscript?	In which section is the information available? (Reagents and Tools Table, Materials and Methods, Figures, Data Availability Section)
Studies involving **human participants**: State details of **authority granting ethics approval** (IRB or equivalent committee(s), provide reference number for approval.	Not Applicable	
Studies involving **human participants**: Include a statement confirming that **informed consent** was obtained from all subjects and that the experiments conformed to the principles set out in the WMA Declaration of Helsinki and the Department of Health and Human Services Belmont Report.	Not Applicable	
Studies involving **human participants:** For publication of **patient photos**, include a statement confirming that consent to publish was obtained.	Not Applicable	
Studies involving experimental **animals**: State details of **authority granting ethics approva**l (IRB or equivalent committee(s), provide reference number for approval. Include a statement of compliance with ethical regulations.	Not Applicable	
Studies involving **specimen and field samples:** State if relevant **permits** obtained, provide details of authority approving study; if none were required, explain why.	Not Applicable	

Dual Use Research of Concern (DURC)	Information included in the manuscript?	In which section is the information available? (Reagents and Tools Table, Materials and Methods, Figures, Data Availability Section)
Could your study fall under dual use research restrictions? Please check biosecurity documents and list of **select agents and toxins** (CDC): https://www.selectagents.gov/sat/list.htm	Not Applicable	
If you used a select agent, is the security level of the lab appropriate and reported in the manuscript?	Not Applicable	
If a study is subject to dual use research of concern regulations, is the name of the **authority granting approval and reference number** for the regulatory approval provided in the manuscript?	Not Applicable	

Reporting

The MDAR framework recommends adoption of discipline-specific guidelines, established and endorsed through community initiatives. Journals have their own policy about requiring specific guidelines and recommendations to complement MDAR.

Adherence to community standards	Information included in the manuscript?	In which section is the information available? (Reagents and Tools Table, Materials and Methods, Figures, Data Availability Section)
State if relevant guidelines or checklists (e.g., **ICMJE, MIBBI, ARRIVE, PRISMA**) have been followed or provided.	Not Applicable	
For **tumor marker prognostic studies,** we recommend that you follow the **REMARK** reporting guidelines (see link list at top right). See author guidelines, under 'Reporting Guidelines'. Please confirm you have followed these guidelines.	Not Applicable	
For **phase II and III randomized controlled trials**, please refer to the **CONSORT** flow diagram (see link list at top right) and submit the CONSORT checklist (see link list at top right) with your submission. See author guidelines, under 'Reporting Guidelines'. Please confirm you have submitted this list.	Not Applicable	

Data Availability

Data availability	Information included in the manuscript?	In which section is the information available? (Reagents and Tools Table, Materials and Methods, Figures, Data Availability Section)
Have **primary datasets** been deposited according to the journal's guidelines (see 'Data Deposition' section) and the respective accession numbers provided in the Data Availability Section?	Not Applicable	
Were **human clinical and genomic datasets** deposited in a public access-controlled repository in accordance to ethical obligations to the patients and to the applicable consent agreement?	Not Applicable	
Are **computational models** that are central and integral to a study available without restrictions in a machine-readable form? Were the relevant accession numbers or links provided?	Not Applicable	
If publicly available data were reused, provide the respective **data citations in the reference list**.	Not Applicable	
